# Study of Bacterial Communities in Water and Different Developmental Stages of *Aedes aegypti* from Aquatic Breeding Sites in Leticia City, Colombian Amazon Biome

**DOI:** 10.3390/insects16020195

**Published:** 2025-02-11

**Authors:** Alejandro Castañeda-Espinosa, Daniela Duque-Granda, Gloria Cadavid-Restrepo, Luz Mila Murcia, Howard Junca, Claudia X. Moreno-Herrera, Rafael J. Vivero-Gómez

**Affiliations:** 1 Grupo de Microbiodiversidad y Bioprospección-Microbiop, Departamento de Biociencias, Facultad de Ciencias, Universidad Nacional de Colombia, St. 65 #59a-110, Medellín 050034, Colombia; acastanedae@unal.edu.co (A.C.-E.); daduquegr@unal.edu.co (D.D.-G.); gecadavi@unal.edu.co (G.C.-R.); 2 Grupo de Estudios en Salud Pública del Amazonas (GESPA), Laboratorio de Salud Pública Departamental del Amazonas, St. 10 #6-127 a 6-1, Leticia 910001, Colombia; lsp@amazonas.gov.co; 3 Microbiomas Foundation, Div. Ecogenomics & Holobionts, RG Microbial Ecology, Metabolism, Genomics & Evolution, LT11A, Chía 250008, Colombia; howard.junca@gmail.com

**Keywords:** *Aedes*, Amazon, microbiota, temperature, salinity

## Abstract

*Aedes aegypti* is a key vector in the transmission of arboviral diseases in the Colombian Amazon. This study aimed to characterize microbiota composition using DNA extracted from water in artificial breeding sites, immature stages, and adults of *Ae. aegypti* in Leticia, Amazonas. Additionally, the physicochemical water variables were correlated with the bacterial communities present. Eight artificial breeding sites were identified, with bucket, plant pot, and tire being the most frequent. The breeding sites exhibited similar physicochemical profiles, with significant temperature and salinity differences (*p*-value < 0.03). The most representative bacterial genera included *Ottowia* (82%), *Xanthobacter* (70.59%), and *Rhodocyclaceae* (92.78%) in breeding site water; *Aquabacterium* (61.07%), *Dechloromonas* (82.85%), and *Flectobacillus* (58.94%) in immature stages; and *Elizabethkingia* (70.89%) and *Cedecea* (39.19%) in males and females of *Ae. aegypti*. Beta diversity analysis revealed distinct clustering between adults and the water and immature communities (*p*-value < 0.001). Multivariate analysis showed strong correlations among bacterial communities, breeding sites, and physicochemical variables such as tire and drum cover which exhibited high levels of total dissolved solids, conductivity, and salinity associated with *Flectobacillus*, *Leifsonia*, *Novosphingobium*, *Ottowia*, and *Rhodobacter*. Bacterial genera such as *Mycobacterium*, *Escherichia*, *Salmonella*, and *Clostridium*, present in artificial breeding sites, are associated with public health relevance. This study provides insights into bacterial community dynamics across *Ae. aegypti*’s life cycle and underscores the importance of water physicochemical and biological characteristics for developing new vector control strategies.

## Simple Summary

The number of arboviral diseases, such as dengue, that are transmitted in the Amazon basin has significantly increased during the last 3 years, with its control becoming more complex as the environmental plasticity and geographical distribution of vectors such as *Aedes aegypti* also increase. Likewise, bacterial communities have favored rapid adaptation to environmental changes at different developmental stages. This problem, from an ecological context, raises the need to generate new knowledge regarding the influence of physicochemical parameters of water and the microbial diversity that are related to the type of artificial breeding sites, and how these interactions can impact the life cycle of *Ae. aegypti.* In the present study, a high richness in bacteria genera from *Ae. aegypti* breeding sites and developmental stages was observed. Some of these genera have biotechnological, entomopathogenic, or antiviral potential. Additionally, we found a close relationship between the bacterial composition profile in the water body and the physicochemical characteristics of the different types of breeding sites. The information derived from this study allowed us to describe the dissolved oxygen, conductivity, and total dissolved solids as key ecological markers associated with the breeding sites from the Amazon basin and highlights the need to understand the microbiome of *Ae. aegypti* for the design of biological control strategies for insect vectors.

## 1. Introduction

*Aedes aegypti* (Linnaeus, 1762) is one of the most significant species worldwide in terms of public health due to its wide distribution and its role as a vector spreading a wide array of viral and parasitic lethal diseases. Particularly critical is the harboring and spreading of arboviruses such as dengue, Zika, chikungunya, Mayaro virus, and yellow fever [1,2]. In recent years, the World Health Organization (WHO) reported 6.5 million cases of dengue, with 80% originating from the Americas and over 7300 deaths attributed to this disease [1,2,3,4].

The Amazon region has historically been recognized for its high prevalence of insect vector-borne diseases, primarily malaria; however, in the Colombian Amazon, the number of cases associated with other vector-borne diseases, such as dengue, has risen significantly from the year 2021 to September 2024, with a total of 2400 cases in the Amazonas department [5,6,7,8], and the circulation of four dengue serotypes, with a higher prevalence of the DENV 2 serotype, during the same period [9,10]. Additionally, the Amazonas department exhibits one of the highest incidence rates of dengue per 100,000 inhabitants, which has increased in recent years [5,6,7,8,9,11]. This trend may be associated with various factors including climatic variability, extreme droughts, human migration dynamics, the agricultural frontier expansion, deforestation, illegal mining, and urbanization, which likely influence and accelerate the environmental plasticity of *Ae. aegypti*, increasing the risk of arboviral disease transmission by arthropods (Arthropod-Borne Virus, ABV) [11].

In Colombia, different insect vector control methods include the use of mosquito nets, repellents, chemical control with insecticides, use of insecticidal paints, and removal of artificial breeding sites [12,13,14,15,16,17]. The implementation of these methods is focused on places with a higher prevalence of cases and with ecological characteristics that potentially increase transmission risk [18,19]. Recently, biological control has also been implemented on a regional scale with the use of endosymbiotic bacteria [20,21,22].

Entomological monitoring for *Ae. aegypti* control frequently includes the detection of breeding sites and the determination of aedic indices to define hotspots associated with social and ecological characteristics that favor a potential higher transmission risk of arboviruses [9,18,19].

Several studies have demonstrated that the types of breeding sites selected by *Ae. aegypti* for oviposition are frequently small and shaded, including tires, drums, buckets, and plant pots, and exhibit characteristic microclimatic conditions of high humidity, commonly containing potable water, although in some cases found with wastewater, brackish water, or rainwater [13,23,24,25]. The eggs hatch in the water and larvae develop in such an environment, where they feed on organic matter associated with algae, microorganisms, and detritus that can impact the size and fecundity of adults, as well as insect vector physiology [13,26]. Physicochemical properties of water such as pH, temperature, salinity, and dissolved solids can modulate bacterial communities in the breeding site water and the larvae, pupae, and adults [24,27]. Similarly, other studies have demonstrated the influence of physicochemical characteristics and their relationship with the presence and abundance of immature stages at mosquito breeding sites [24,28,29,30,31].

However, the bacterial communities present in the breeding sites remain an underexplored area of study, despite their significant importance in the ecology and vector competence of *Ae. aegypti.* Hery et al. described the bacterial communities associated with artificial breeding sites and immature stages of *Ae. aegypti* in Guadeloupe and French Guiana. They compared the physicochemical characteristics and bacterial abundance, observing a positive correlation between higher values of conductivity, calcium (Ca), magnesium (Mg), and dissolved oxygen, and a higher abundance of *Acinetobacter*, *Pseudomonas*, *Gemmobacter*, *Polynucleobacter*, and *Ancylobacter* bacteria. They also highlighted a lower relative abundance of *Bacillus* and *Herbiconiux*.

Studies about the *Ae. aegypti* microbiome more often include mosquito adults and the influence of variables that can affect vectorial capacity, such as thermotolerance [32,33] and insecticide resistance [34,35]. In recent decades, diverse culture-dependent and -independent methods have been conducted to characterize the microbial communities of different organs and tissues (gut, reproductive system, salivary glands) [34,35,36,37,38]. In these studies, bacteria of the genera *Asaia*, *Bacillus*, *Acinetobacter*, *Elizabethkingia*, *Enterobacter*, *Pantoea*, *Pseudomonas*, and *Serratia* are most commonly reported in association with *Ae. aegypti* adults [36,37,38,39,40]. These genera are also reported in immature stages of *Ae. aegypti*, in addition to other bacteria like *Stenotrophomonas* and *Microbacterium*, while a higher genera diversity has also been found in early mosquito developmental stages [41,42,43,44].

Research on the *Ae. aegypti* microbiome has provided new insights into several important factors, including insecticide resistance, the degradation of various carbon sources (xylose, glucose, blood, nectar), fitness, and immune response. These factors are particularly crucial in the context of arbovirus transmission [10,40,45,46,47,48]. For instance, certain species of *Serratia* bacteria like *S. odorifera* and *S. marcescens* have been found to play a beneficial role in the establishment of the dengue virus, serotype 2 (DENV-2), and Zika virus (ZIKV) in *Ae. aegypti*, through protein excretion that suppresses the mosquito’s immune response favoring its vector competence [45,49,50]. Over the last decade, there has been growing interest in the potential use of secondary endosymbionts or commensal bacteria for biological control of arboviruses, including *Wolbachia* and *Rosenbergiella* [10,22,51,52]. In particular, observations on *Rosenbergiella* bacteria show that their action through enzyme production to metabolize blood alters the midgut pH, inactivating viral particles, representing an alternative strategy for biological control [22].

An alternative approach to controlling insect vectors involves studying the physicochemical parameters and exploring the bacterial communities associated with *Ae. aegypti* populations in the Amazon, considering its biotechnological potential for control. Given the high diversity of these populations and the limited research conducted in the region, this exploration could lead to valuable insights regarding *Aedes* breeding sites. It may improve the characterization of potential spots with a higher risk of transmission and space–time modeling, which is essential for predicting future outbreaks of ABV epidemics [25].

Considering the local and global circumstances related to the increase and lethality of ABV cases in Colombia, as well as the challenges faced in disease control and its impact on public health especially in strategic areas like the Colombian Amazon, which is influenced by factors of eco-epidemiological complexity such as climate change and ongoing fauna and human migration, this study aimed to characterize the bacterial composition present in the water of breeding sites and the different developmental stages of *Ae. aegypti* from Leticia, Amazonas. Additionally, we correlated the physicochemical parameters of these water bodies with the bacterial communities identified.

## 2. Materials and Methods

### 2.1. Collection Permits

Insect collection in the Amazonas department was conducted under the Framework Permit for Collecting Specimens of Wild Species of Biological Diversity for Non-commercial Scientific Research Purposes, conceded to the Universidad Nacional de Colombia by the Resolution 0255 of 12 March 2014 of the Autoridad Nacional de Licencias Ambientales. Initially, we explained to the community from the targeted neighborhoods the context and importance of insect vector control and the phases of the project. We then proceeded to take samples from public areas and private properties after obtaining consent from landowners.

### 2.2. Collection Area

**Figure 1:**
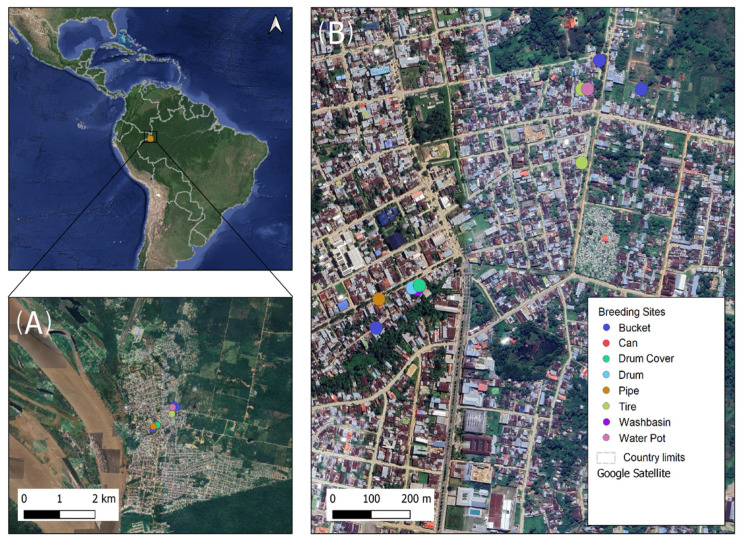
Sampling area satellite view indicating the positive breeding sites identified with *Ae. aegypti.* (**A**) View of Leticia, Colombia, and its national borders. (**B**) Location and type of positive artificial breeding sites (in circles) of *Ae. aegypti* were detected during an extreme dry season in November of 2023.

Samples were collected in the municipality of Leticia, Amazonas department, Colombia, and the area was determined in collaboration with the Laboratorio de Salud Pública del Amazonas (L.S.P.A.) considering neighborhoods with the highest number of reported cases of arboviruses during the 2023 period. Entomologic exploration was conducted inside dwellings and in the peridomestic areas of Once de Noviembre (4.2122° S, 69.93465° W), Colombia (4.21816° S, 69.93451° W), and Porvenir (4.2191° S, 69.93831° W), neighborhoods of Leticia, Amazonas (Figure 1). These neighborhoods are adjacent to the Brazilian border and between 1 and 2 km near the Amazon River, at an approximate altitude of 80 m above sea level (m.A.S.L). The mean temperature during the sampling period was 31 °C, with minimum and maximum temperatures of 23 °C and 37 °C, respectively, and a relative humidity of 90%, according to the meteorological station at the Alfredo Vásquez Cobo International Airport [53], as well as precipitations of 150 mm [54].

### 2.3. Search of Breeding Sites and Physicochemical Analysis of Water Samples Collected at Ae. aegypti Artificial Breeding Sites

The search for breeding sites was conducted using previously described methodologies with certain modifications [24,30]. A visual inspection of containers with stagnant water in the peridomestic forecourt, courtyard, and gardens of dwellings was performed for 6 h/day during three days with three people to identify positive breeding sites, targeting culicid larvae based on their movement and siphon size [24].

**Figure 2:**
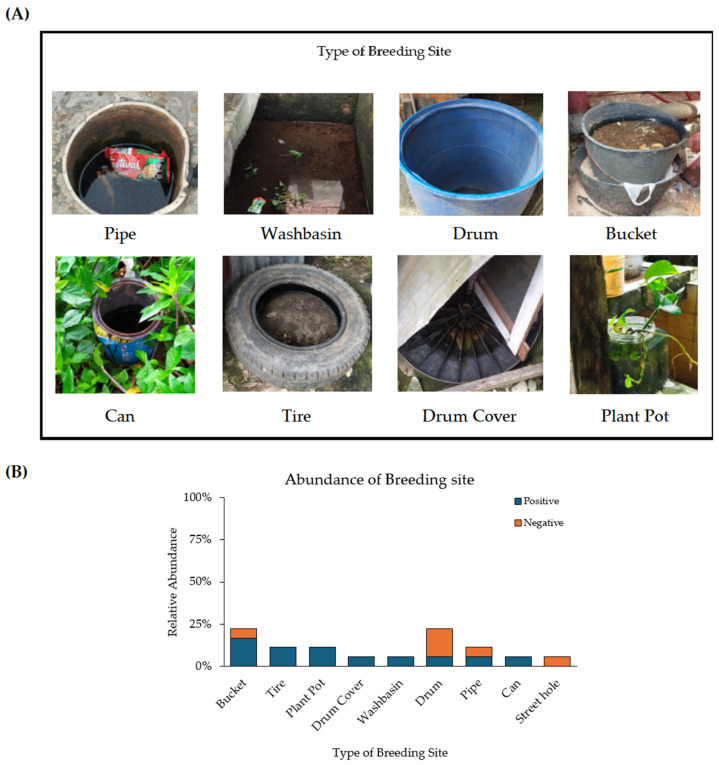
(**A**) Type of positive artificial breeding sites for *Ae. aegypti* found in Leticia, Amazonas. (**B**) Relative abundance of positive and negative breeding sites.

Following the active search and confirmation of positive artificial breeding sites by the presence of immature culicids, specifically *Ae. aegypti*, which was the only species associated with the sampled breeding sites in Leticia, photographic records were taken and a general description of the breeding sites was performed. These were classified into 9 categories (1–3 breeding sites per classification): (I) washbasin, (II) bucket, (III) drum, (IV) can, (V) tire, (VI) plant pot, (VII) drum cover, (VIII) pipe, and (IX) street hole (Figure 2, Table S1). Subsequently, water characteristics were measured in situ using a ProDSS multiparameter probe (YSI, USA), including temperature (°C), pH, barometric pressure (mmHg), coordinates, dissolved oxygen percentage (%DO), oxygen concentration (mg/L), electrical conductivity (μS/cm), total dissolved solids (mg/L), and salinity (ng/L). Then, an ANOVA and Tukey test were performed (see Section 2.7). Finally, after classified breeding sites and statistical analysis, larval housing, storage, and Breteau indexes were calculated.

### 2.4. Sample Collection and Total DNA Extraction from Artificial Breeding Site Water for the Study of the Bacterial Communities Associated with the Different Aedes Artificial Breeding Site Types

After estimating the physicochemical parameters at each breeding site, 37.5 mL of water samples was collected using a 20 mL Pasteur pipette and deposited into sterile 50 mL plastic tubes. Subsequently, 12.5 mL of Longmire’s solution (100 mM Tris; pH 8.0, 100 mM EDTA; pH 8.0, 10 mM NaCl, 0.5% SDS, 0.2% sodium azide) was added to preserve the samples, which were homogenized by inversion [55].

Then, the bacterial communities and other microorganisms were retained on 0.22 μm filters (Whatman 25 mm). Approximately 20 mL of water sample was filtered, after which the filter was replaced using sterile tweezers and deposited into 5 mL tubes [56]. DNA extraction was performed using the DNeasy PowerWater kit (Qiagen, Hilden, Germany) following the manufacturer’s protocol. DNA quantification was conducted using a Nanodrop spectrophotometer (IMPLEN, Munich, Germany). Samples were subsequently concentrated and stored at –20 °C for use in PCR amplification of the 16S rRNA gene and sequenced to study the bacterial communities associated with artificial breeding sites (see Section 2.7).

### 2.5. Collection of Immature Aedes and Taxonomic Identification of Ae. aegypti Mosquitoes

Pasteur pipettes and plastic dippers were used to collect immature *Aedes* from breeding sites, which were placed in a container covered with muslin and filled with water from the breeding site. This allowed for the collection of immature (larvae and pupae) and adult *Aedes* associated with different breeding site types. The immature *Aedes* were transported to the L.S.P.A. under insectary conditions and reared at 28 °C with 80% relative humidity and a 12:12 light–dark photoperiod [57].

A proportion of larvae (L1−L2, L3−L4) and pupae of each breeding site (bucket, plant pot, and tire) were preserved with ethanol at −20 °C, while the other proportion was used to obtain specimens of males and females. Larvae and pupae were raised to adulthood in muslin cages in the same water where the larvae were collected. Once the adults emerged (one day old), they were individualized in collection flasks and cold-sacrificed at −20 °C, avoiding copulation and possible modification of the bacterial communities by feeding habits. Adults were not fed sucrose solution either.

Specimens (L3−L4 larvae and adults of both sexes) from each breeding site were morphologically classified using taxonomic keys from González et al. [58] under a stereoscope (Motic, Xiamen, China). Subsequently, groups of immature *Aedes* (L1−L2, L3−L4, pupae) and adults (males and females) were formed depending on availability from each breeding site (Table S2). A total of 57 individuals of *Ae. aegypti* were used in the present study: L1/L2 (3 pools each with 5 larvae), L3/L4 (3 pools with 1–5 larvae), pupae (3 pools with 1–5 pupae), males (2 pools with 4–5 individuals), and females (2 pools with 4–5 individuals)**.** These samples were prepared for DNA extraction and bacterial community sequencing using Illumina Miseq technology (see Section 2.6).

As an external control and reference pattern, adults of *Ae. albopictus* were used. They were also obtained from eggs collected through ovitraps. The ovitraps were disposed of after seven days in the domicile and peridomicile of the study area. Then, the paper towels with oviposition were transported to the L.S.P.A. and air-dried for 24 h. After that, the egg papers were placed into a plastic container where the eggs hatched, and larvae were reared until adults were obtained. Finally, 10 *Ae. albopictus* adults (5 individuals per pool) were selected and processed for DNA extraction (see Section 2.6). It is important to note that due to low abundance during the collection timeframe, *Ae. albopictus* is considered just as an external control, given that *Ae. aegypti* and *Ae. albopictus* coexisted in the study area.

### 2.6. DNA Extraction from Immature and Adult Aedes from Artificial Breeding Sites for the Analysis of Bacterial Communities

Total DNA extraction from immature and adult *Ae. aegypti* from artificial breeding sites were conducted using the Quick-DNA Tissue/Insect Miniprep Kit (Zymo-Research, Irvine, CA, USA) with slight modifications. Initially, larvae, pupae, and adults were washed individually with ethanol 70% *v*/*v*. Then, the ethanol was removed, and insects were air-dried and pooled at 5 individuals per vial. Subsequently, 300 μL of “BashingBead™ Buffer” was added, and the samples were macerated with a micropestle until fully homogenized [59]. After manual maceration, an additional 450 μL of “BashingBead™ Buffer” was added. To optimize homogenization, half a column of glass beads were added to each vial, followed by vortexing and cold bath cycles for 10 min: 3 min of vortexing and 3 min at −20 °C. The protocol was then continued as per the manufacturer’s recommendations. DNA was eluted by adding 50 μL of “DNA Elution Buffer” and centrifuging at 10,000× *g* for 30 s. An additional 50 μL was added, followed by a second centrifugation. Extracted DNA was analyzed with a Nanodrop spectrophotometer (Implen, Munich, Germany), yielding DNA samples with an absorbance ratio (280 nm/260 nm) within the range of 1.8 to 2.0.

This biological material was used for PCR amplification of the 16S rRNA gene and sequenced to study bacterial communities (see Section 2.7). Extraction controls included 70 μL aliquots of Core Community (Zymo-Research, Irvine, CA, USA) and ultrapure, autoclaved, irradiated Type I water.

### 2.7. Amplification, Paired-End Illumina Sequencing, and Bioinformatic and Statistical Analyses

Conventional PCR targeting of the 16S rRNA gene was performed to verify the presence of bacterial DNA. Primers 27F (5′-AGAGTTTGATCCTGGCTCAG-3′) and 1492R (5′-GGTTACCTTGTTACGACTT-3′) were used following the conditions and thermal profile described by Espejo et al. [60]. Amplicons were visualized using 1.2% agarose gel electrophoresis at 80 V for 45 min.

The estimation of fragment sizes and DNA quantities was performed using a Bioanalyzer. The results showed that 60% of the detected fragments had an average size >800 bp, with total DNA extraction yields surpassing 40 ng per processed sample.

For PCR amplification of the V3–V4 hypervariable regions of the 16S rRNA gene, 10 ng of metagenomic DNA from each sample was used as a template. This resulted in a PCR product with an average length of 470 bp. The primers used were 341F (5′-CCTAYGGGRBGCASCAG-3′) and 806R (5′-GGACTACNNGGGTATCTAAT-3′) [61]. The reaction was performed using a Phusion High-Fidelity PCR Master Mix (Biolabs, Ipswich, MA, USA) under previously optimized conditions. Each reaction contained 250 picomoles of each primer and 3% DMSO. The thermal cycling conditions included an initial denaturation at 98 °C for 2 min, followed by 35 cycles of denaturation at 98 °C for 15 s, annealing at 55 °C for 15 s, extension at 68 °C for 30 s, and a final extension for 5 min. Libraries compatible with Illumina sequencing platforms were constructed from the amplification products using the NEBNext Ultra™ II DNA PCR-Free Library Prep Kit (New England Biolabs) and sequenced on an Illumina HiSeq 2500 PE250 platform.

The obtained average reads per sample were 205,428 using 250 cycles on each paired-end direction. The raw paired-end Sequence Read Archive (SRA) datasets are available at NCBI repository Bioproject PRJNA1185382 on BioSamples SAMN44702301 to SAMN44702308 for water samples; meanwhile, whole-body insect samples are available on BioSamples SAMN44731586 to SAMN44731600.

Amplicon Sequence Variant (ASV) classification was conducted to assess the frequency of bacterial genera and species in the samples. The DADA2 pipeline (version 1.16) was used for quality filtering, read assembly, chimera removal, and taxonomic assignment. Taxonomic classification of ASVs was performed using the RDP Classifier with the SILVA 138.1 database and RDP for higher taxonomic ranks. Filtering parameters included the following: “*out <- filterAndTrim(fnFs, filtFs, fnRs, filtRs, compress = TRUE, truncQ = 2, truncLen = c(226,226), trimLeft = c(1,1), maxN = 0, maxEE = c(2,2), rm.phix = TRUE, matchIDs = TRUE, multithread = TRUE)*”.

The processing yielded an average of 182,045 merged sequences per sample, with a minimum of 166,309 and a maximum of 192,597 reads.

Following taxonomic annotation and removal of non-relevant ASVs (e.g., chloroplasts, mitochondrial DNA), additional curation was performed for the top 150 most frequent ASVs to define whether there is a higher taxonomic resolution for the ASVs that did not reach a species-level annotation with SILVA 138.1; we performed a similarity search against the 16S dataset of the type of species reported, with updates in LPSN (https://lpsn.dsmz.de/), EzBioCloud (https://www.ezbiocloud.net/), and BlastN searches (https://blast.ncbi.nlm.nih.gov/) of the NCBI 16S ribosomal RNA database for bacteria- and archaea-type strains (sites accessed on 4 November 2024). This approach allowed improved taxonomic resolution for certain ASVs, with species-level annotations included where possible.

All of the ASVs removed were of very low frequency, indicating high specificity and efficiency of the amplification for the target 16S bacterial sequences. The most frequent ASVs showed close matches to reference bacterial-type species, ensuring accurate taxonomic assignments.

The results were analyzed using the online platform MicrobiomeAnalyst [61,62,63] to generate rarefaction curves and relative abundance plots at the phylum, genus, and species levels. Additionally, the core microbiome was identified and a heatmap was created. The same platform was used to estimate α-diversity (Chao1, Shannon, and Simpson indices) through analysis of variance (ANOVA) and β-diversity via Bray–Curtis dissimilarity using principal coordinates analysis (PCoA) and non-metric multidimensional scaling (NMDS). A network interaction analysis was also performed using Pearson correlation coefficients.

To evaluate the influence of physicochemical variables on positivity and differences among breeding site types, a one-way ANOVA was performed, followed by Tukey’s test for group comparisons and paired analyses with a 95% confidence level (*p*-value ≤ 0.05), implemented in R v.4.4.1. A gbitplot was created to visualize the influence of physicochemical variables on breeding site types. Additionally, in PAST v4.17 (PAleontological STatistics, Version 4.17), a multivariate analysis using Principal Component Analysis (PCA) was conducted to explore potential correlations between bacterial communities from different water bodies and their physicochemical variables. This analysis focused on the 20 most abundant bacteria genera and their relationships with physicochemical parameters of different breeding site types, based on the Bray–Curtis index.

### 2.8. Study Limitations

Certain limitations were encountered within this research, particularly during sample collection. Climatic conditions during this period in the Amazonas department included one of the most severe droughts on record, with an average temperature of 31 °C, maximums reaching 37 °C [53], and low precipitation levels [54]. These conditions likely contributed to the reduced availability of breeding site types and the potential absence of *Ae. albopictus*, as well as the development of breeding sites even in containers with direct sun exposure and high levels of leachate materials.

## 3. Results

### 3.1. Description and Classification of Ae. aegypti Artificial Breeding Sites

After actively searching for positive breeding sites in Leticia, larval indexes such as larval housing (26.6%,) storage (66.6%), and Breteau (52.2%) were calculated. Also, 12 positive culicids breeding sites and 6 negative ones with no immature *Aedes* were identified (Figure 2). The identified immature *Aedes* were taxonomically classified as *Ae. aegypti*. Also, ten adults of *Ae. albopictus* were collected only using ovitraps. Due to their low abundance, these adults were used as an interspecific comparison group.

A total of 18 breeding sites were identified in both peridomestic and dwelling areas, categorized into nine different types based on the type of container and their dimensions: (I) washbasin (6%), (II) bucket (22%), (III) drum (22%), (IV) can (6%), (V) tire (11%), (VI) plant pot (11%), (VII) drum cover (6%), (VIII) pipe (11%), and (IX) street hole (6%) (Figure 2, Table S1). Most of these breeding sites were located in high-humidity microenvironments, without direct sunlight exposure, such as the buckets, drums, plant pots, washbasins, tires, and drum cover. However, the pipe, can, and one of the tires analyzed were found in open fields or on the street with continuous sun exposure. These particular breeding sites presented higher temperatures (30 to 33 °C, Table S3) and different microclimatic conditions due to the lack of surrounding vegetation and direct sunlight exposure compared to the other breeding sites.

In addition, the observed characteristics of the water, such as turbidity and the presence of sediments, do not coincide with previous reports associated with artificial breeding sites, that is, the water of the positive breeding sites sampled in Leticia, Amazonas, was observed with sand or small solid objects suspended in the same material as the container, such as the can, washbasin, and bucket (Figure 2). It is important to highlight that representations of specimens from all stages of the life cycle were obtained by type of breeding site. Nevertheless, the relative abundance of immature specimens was not calculated, because the aim of this study focuses mainly on the physicochemical and bacterial community characterization of breeding sites and the different developmental stages of mosquitoes, which allowed us to establish a small experimental size, indicating that this study is neither spatiotemporal nor systematic, to represent variations in the abundance of immature specimens by type of breeding site and other types of correlations.

### 3.2. Characterization of the Physicochemical Variables of Artificial Breeding Sites of Ae. aegypti

**Figure 3:**
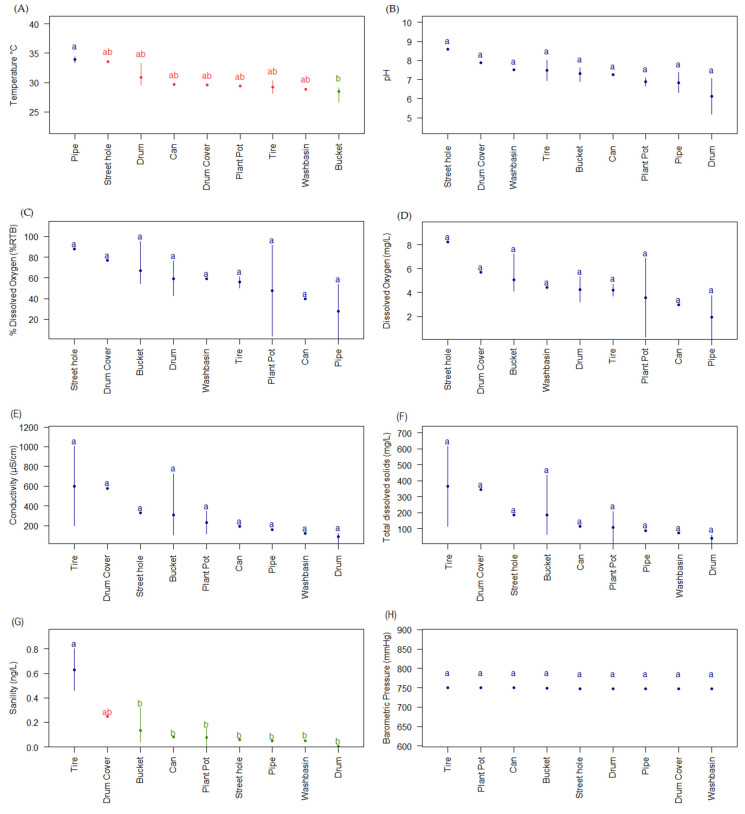
Physicochemical characteristics of *Ae. aegypti* artificial breeding sites grouped by Tukey’s statistics. (**A**) Temperature (*p*-value = 0.028); three groups (a, ab, and b) are shown that are significantly different. (**B**) pH (*p*-value = 0.17). (**C**) Dissolved oxygen percentage (*p*-value = 0.73). (**D**) Dissolved oxygen concentration (*p*-value = 0.49). (**E**) Electrical conductivity (*p*-value = 0.53). (**F**) Total dissolved solids (*p*-value = 0.51). (**G**) Salinity (*p*-value = 0.008); three groups (a, ab and b) are shown with statistically significant differences. (**H**) Barometric pressure (*p*-value = 0.056).

The physicochemical profile of *Ae. aegypti* artificial breeding sites, specifically temperature and salinity, showed significant variations among them. The temperature of the breeding sites (ANOVA, F-value = 3.96 *p*-value = 0.03) was recorded at an average value of 29 °C (sd = 1.56), with a maximum value of 33.4 °C associated with the pipe with direct exposure to the sun (Group a, Figure 3A), and a minimum of 26.6 °C related to the bucket, located under a roof and surrounded by vegetation (group b, Figure 3A). The temperature in the other types of breeding sites was similar in terms of the average and did not present significant differences (Group ab, Figure 3A).

On the other hand, salinity (ANOVA, F-value = 5.76, *p*-value = 0.008) showed variations among the breeding sites, revealing three statistically different groups among them. The first group, represented by Group a (Figure 3G), was associated with the tire, with an average value of 0.63 ng/L (sd = 0.24), which was in an area of inadequate waste disposal, where it was exposed to air, solar radiation, and possible leachates from other waste that could influence the values of this parameter. A second group was made up of the drum cover breeding site (Group ab, Figure 3G) with a value of 0.25 ng/L (Table S3), which contained rainwater that had possibly been reduced due to evaporation, increasing the salt concentration present in the container. Finally, a third group, Group b (Figure 3G), comprising a bucket, can, plant pot, street hole, washbasin, pipe, and drum breeding site, had an average salinity range of 0.005–0.13 ng/L (sd = 0.08) (Figure 3G). The above may suggest that *Ae aegypti* larvae and pupae present plasticity through physicochemical characteristics such as temperature and salinity found in the different breeding sites in Leticia, Amazonas.

It was observed that the other physicochemical parameters were conserved (ANOVA, range of F-value = 0.6 to 3.1 and *p*-value > 0.05, Table S3), which suggests that they may be important ecological markers for the selection of *Ae. aegypti*, such as pH (average value = 7.1, sd = 0.42, *p*-value = 0.17), dissolved oxygen (average value = 57.3%, sd = 21.21, *p*-value = 0.73 or 4.3 mg/L, sd = 1.60, *p*-value = 0.49), conductivity (average value = 265.8 μS/cm, sd = 299.78, *p*-value = 0.53), total dissolved solids (average value = 152.7 mg/L, sd = 187.83, *p*-value = 0.51), and barometric pressure (average value = 748.7 mmHg, sd = 1.55, *p*-value = 0.056), which did not show significant differences among the different sampled breeding sites (Figure 3B–F,H, Table S3).

**Figure 4:**
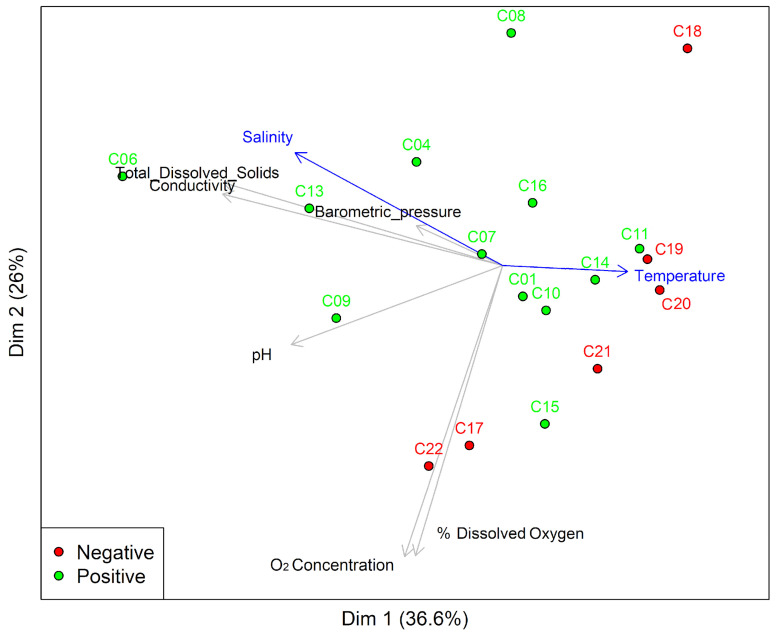
Bitplot representation of the PCA of the relationship between physicochemical variables and positive/negative categorization of *Ae. aegypti* breeding sites. Vector lines represent physicochemical parameters and the direction head of the vectors indicates an increase in magnitude. The points and codes refer to the positive (green color) and negative (red color) breeding sites.

Principal Component Analysis (PCA) represented 62.2% of the variance in terms of the influence of the physicochemical variables of the artificial breeding site on the presence (positive) or absence of immature *Aedes* (negative) (Figure 4) and presented statistically significant differences associated with temperature (ANOVA F-value = 6.81 and *p*-value = 0.01) (Figure S1). The other physicochemical characteristics remained similar between them. It is essential to emphasize salinity, total dissolved solids, conductivity, and pH, as these factors may have a more significant influence (Figure 4, Table S3).

### 3.3. Bacterial Communities of Water Bodies from Artificial Breeding Sites and Developmental Stages of Ae. aegypti

After analyzing twenty-four samples, 3,352,520 filtered sequences were obtained, ranging from 30,723 to 188,684 per sample (Table S4). The rarefaction curve shows a high number of reads and taxa, demonstrating good coverage of bacterial communities and an adequate sampling effort, as evidenced by the plateau seen in most samples (Figure S2). A total of 6893 ASVs were obtained with an average of 861.625 ASVs per water sample and 3212 were detected for different *Ae. aegypti* developmental stages, with an average of 214.13 per sample.

#### 3.3.1. Richness and Alpha Diversity of the Water Bacterial Community of Artificial Breeding Sites and Developmental Stages of *Ae. aegypti*

**Figure 5:**
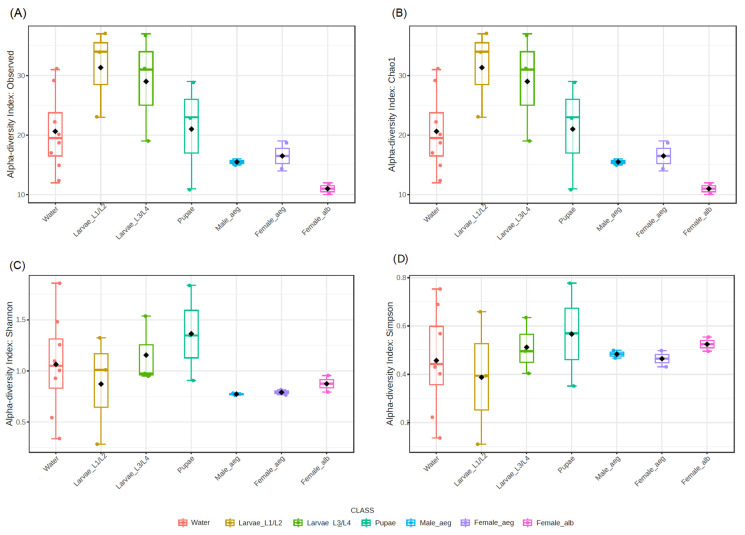
ASV richness and alpha diversity indices of water from the artificial breeding sites and *Ae. aegypti* and *Ae. albopictus*. (**A**) Observable richness (F-value = 2.02; *p*-value = 0.12), (**B**) Chao 1 index (F-value = 2.02; *p*-value = 0.12), (**C**) Shannon index (F-value = 1.01; *p*-value = 0.45), and (**D**) Simpson index (F-value = 0.53; *p*-value = 0.78). Water, water samples from artificial breeding sites; Larvae_L1/L2, samples of L1–L2 stage larvae; Larvae_L3/L4, samples of L3–L4 stage larvae; pupae, samples of pupae; Male_aeg, males of *Ae. aegypti*; Female_aeg, females of *Ae. Aegypti*; and Female_alb, females of *Ae. albopictus*. The black rhombus represented the mean value and the middle whiskers in the box were the median.

The observed microbial richness showed a higher number of genera in L1–L2 larvae and water samples than in later developmental stages, which decreased as the larvae matured into adults (Figure 5A). The Chao 1 (F-value = 2.02; *p*-value = 0.12), Shannon (F-value = 1.01; *p*-value = 0.45), and Simpson (F-value = 0.53; *p*-value = 0.78) indices did not show significant differences between the different groups. However, it was possible to identify that the adults of *Ae. aegypti* and *Ae. albopictus* presented lower richness and alpha diversity. Even between them, the females of *Ae. albopictus* were the group with the lowest richness and diversity (Figure 5).

#### 3.3.2. Bacterial Communities from Breeding Site Water and Developmental Stages of *Ae. aegypti*

**Figure 6:**
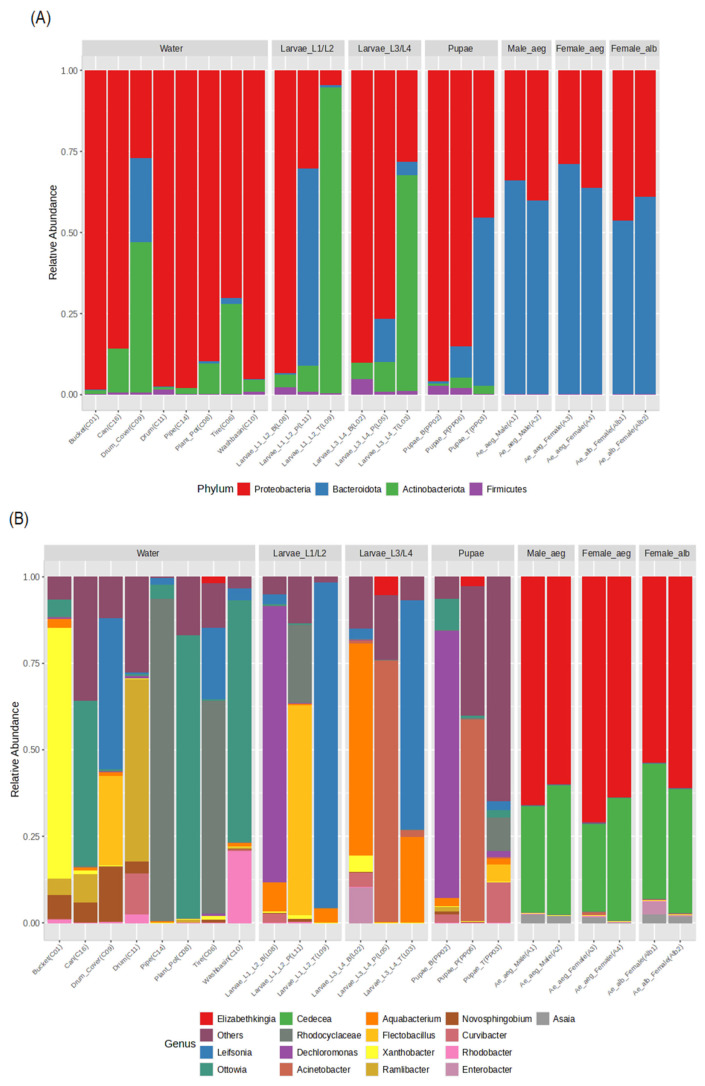
Relative abundance profiles of ASVs from water bodies of artificial breeding sites, larvae, pupae, and adults of *Ae. aegypti* and *Ae. albopictus* females. (**A**) Relative abundance at the phylum level. (**B**) Relative abundance of principal genera.

Phyla Proteobacteria (4.56–95.76%), Actinobacteriota (0.77–94.40%), and Bacteroidota (0.08–71.29%) were found with different abundances and frequencies depending on the source sample (Figure 6A). The bacterial communities of water bodies presented a high abundance of Proteobacteria (>73.31%) and Actinobacteriota (0.08–25.19%) and a lower proportion of Bacteroidota (0.05–25.71%), except for the drum cover (C09) (25.84%). The groups of *Ae. aegypti* exhibited more evenness in the abundance at the phylum level (Figure 6A). However, pupal samples had a higher representation of Proteobacteria (>85%), except for Pupa_p (PP06), where Bacteroidota predominated (52%). In the adult stages of *Ae. aegypti* and *Ae. albopictus*, the relative abundance was mainly dominated by Bacteroidota (53.89–71.29%) (Figure 6A).

At the genus level, high richness and variability of ASVs were presented according to the types of artificial breeding sites, highlighting the following findings: tires, plant pots and cans were dominated by *Ottowia* (47.75–82–39%), *Xanthobacter* was found in buckets (70.59%), *Rhodocycloceae* was found in washbasin and pipes (49.19 and 92.78%), *Ramilibacter* was found in drums (53.08%) and in the drum cover there was greater equality between *Novosphingobium* (15.84%), *Flectobacillus* (25.56%), and *Leifsonia* (43.42%) (Figure 6B). A different composition of bacterial genera was detected between *Ae. aegypti* stages, also indicating high intraspecific richness in the larval groups (L1−L2, L3−L4) and even in pupae, which may be associated with the type of breeding site they specifically came from. The L1−L2 larvae presented a high abundance of the genera *Leifsonia* (94.20%), *Dechloromonas* (82.85%), and *Flectobacillus* (58.94%), while the L3−L4 larvae were dominated by *Aquabacterium* (61.07%) and *Acinetobacter* (75.47%) (Figure 6B). The pupae exhibited a high presence of *Dechloromonas* of 77.95%, followed by *Acinetobacter* (60.65%) and *Chryseobacterium* (33.39%) (Figure 6B). In contrast, the adults of *Ae. aegypti* and *Ae. albopictus* showed a significant abundance of *Elizabethkingia* (53.88–70.89%), *Cedecea* (25.26–39.19%), and *Asaia* (1.20–2.60%) (Figure 6).

Interestingly, *Elizabethkingia* with abundances between 1 and 2% in larvae could be acquired from some artificial breeding sites (Figure 6); it was also established in pupae with similar abundances, and was seen in abundance for the *Ae. aegypti* adult stage, exceeding 50%. Other ASVs, such as *Acinetobacter*, *Chryseobacterium*, and *Aquabacterium* (Table S5), were found in all developmental stages and water samples, suggesting a possible transmission through life cycle stages.

**Figure 7:**
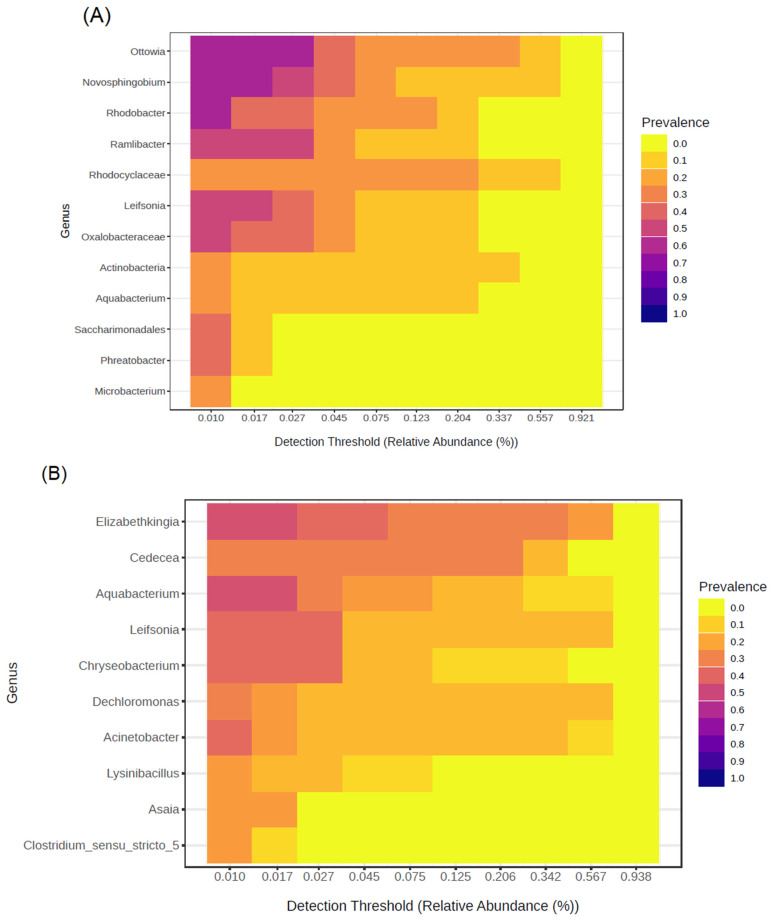
The core community of water samples from artificial breeding sites of immature and adult *Ae. aegypti*. (**A**) Core community of water bodies from artificial breeding sites for *Ae. aegypti*. (**B**) The core community of immature and adult *Ae. aegypti.* Twelve bacterial genera were observed associated with artificial breeding sites and eight were associated with immature and adult *Ae. aegypti*, with prevalences of 20–60% and relative abundances less than 0.567%.

The core community related to water bodies from artificial breeding sites differs between immature and adult *Ae. aegypti*. In water samples collected from these breeding sites, twelve bacterial genera were identified. The most prevalent were *Ottowia*, *Rhodobacter*, and *Novosphingobium*, which together accounted for 63% of the samples (Figure 7A). Regarding the immature and adult *Ae*. *aegypti*, eight bacterial genera were identified as being part of their core community. Notably, *Elizabethkingia* and *Aquabacterium* were the most prevalent, found in 46% of the samples (Figure 7B). Another important finding was that the genera *Aquabacterium* and *Leifsonia* both had a prevalence of 50% in the core community of the water bodies. In the case of immature and adult *Ae*. *aegypti*, *Aquabacterium* was present in 46% of the samples, while *Leifsonia* was found in 38% (Figure 7).

A pattern search using the Pearson correlation coefficient (r) allowed us to establish positive and negative correlations among the most abundant bacterial genera at different developmental stages. The genus *Asaia* presented positive correlations with *Cedecea* and *Elizabethkingia* (r = 0.88–0.87; *p*-value = 2.78 × 10^−8^–4.94 × 10^−8^; FDR = 1.03 × 10^−6^–1.22 × 10^−6^), which were strongly correlated with the adult stages of *Ae. aegypti* and *Ae. albopictus* (Figure S3, Table S6). Additionally, negative relationships were found between *Asaia* and genera *Microbacterium* (r = −0.34), *Stenotrophomonas* (r = −0.28), and *Bacillus* (r = −0.28), without statistically significant differences (*p*-value > 0.05, FDR = 0.59). These genera were predominantly associated with breeding site water samples, pupae, and L3–L4 larvae (Figure S3, Table S6). In general, the genus with the highest abundance and correlations associated with the bacterial communities in water samples included *Aeromicrobium* and *Ottowia*, Larvae L1–L2 was related to *Flectobacillus*, L3–L4 was related to *Aquabacterium*, and pupae were linked to *Acinetobacter*, *Chryseobacterium*, and *Dechloromonas*. These genera exhibited a tendency for negative correlations with *Asaia*, *Cedecea*, and *Elizabethkingia*, which were strongly related to the adult stages (Figure S3), although the differences were not statistically significant.

**Figure 8:**
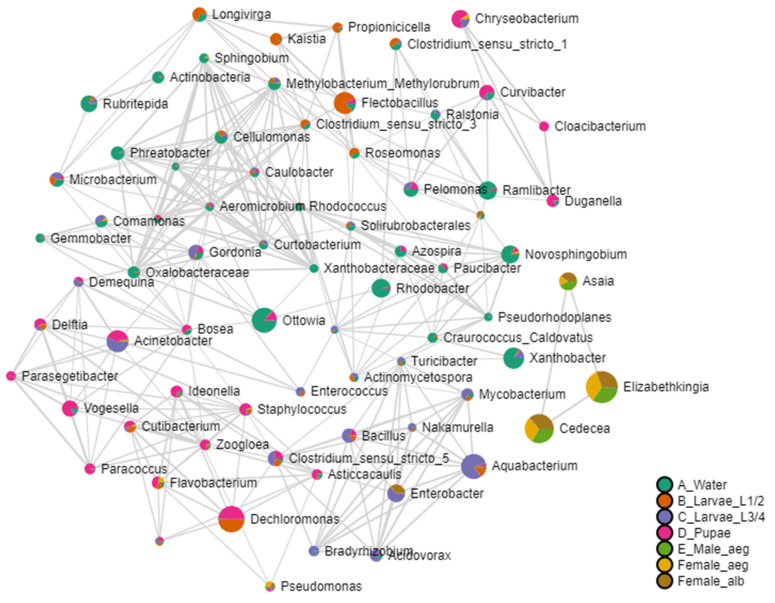
Interaction network between the most abundant genera using the Pearson correlation coefficient (r) at the genus level of the total samples analyzed. The network presents 6 different groups separated by the bacterial genera that correlate with the different samples: breeding site water (green), Larvae L3–L4 (purple), pupae (pink), and adults (yellow). The group that includes the bacterial genera most related to Larvae L1–L2 is shown in orange. Water, samples of water from artificial breeding sites; Larvae_L1/2, samples of larvae at the L1–L2 stage; Larvae L3/4, samples of larvae at the L3–L4 stage; pupae, samples of pupae; Male_aeg, males of *Ae. aegypti*; Female_aeg, females of *Ae. aegypti*; and Female_alb, females of *Ae. albopictus*.

The interaction network established using the Pearson coefficient revealed an organization of six distinct groups of bacterial communities, categorized by their developmental stage or origin (Figure 8). *Ae. aegypti* breeding site water samples, located in the center of the network, showed correlations with all of the immature stages, highlighting the genera related to L1–L2 larvae (Figure 8). Nonetheless, the pupae presented two groups, one related to the water samples and the other integrating correlations between water samples and L3–L4 larvae. Additionally, a separate group was observed consisting of bacterial genera *Asaia*, *Cedecea*, and *Elizabethkingia*, mainly related to the adult stages.

#### 3.3.3. Beta Diversity of Bacterial Communities in Artificial Breeding Water and Immature and Adult *Ae. aegypti*

**Figure 9:**
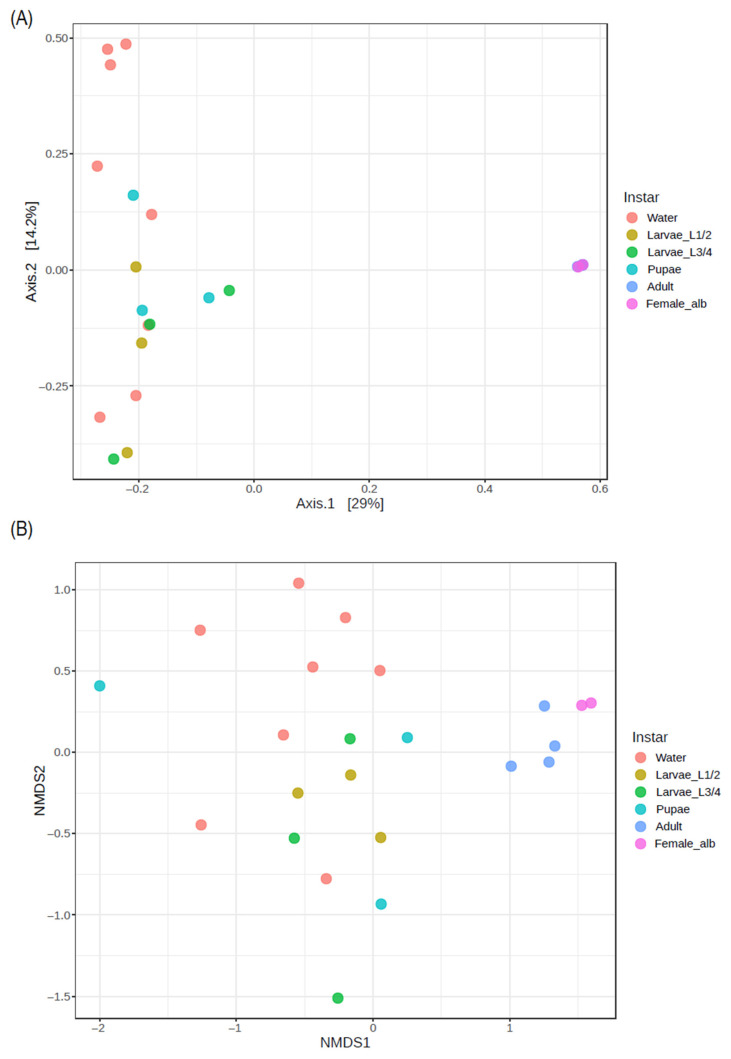
Beta diversity of bacterial communities in artificial breeding site water samples, larvae, pupae, and adults of *Ae. aegypti* and females of *Ae. albopictus*. (**A**) Principal coordinates analysis (PCoA) was generated with the Bray–Curtis index (PERMANOVA F-value = 2.60; *p*-value = 0.001; R^2^ = 0.43). (**B**) Nonparametric multidimensional scaling (NMDS) analysis: significant differences were found between adults of *Ae. aegypti* and water body samples from breeding sites, larvae, and pupae forming two different groups (PERMANOVA F-value = 2.60; *p*-value = 0.001; R^2^ = 0.43 and a stress level of 0.19). Water: water breeding sites samples; Larvae_L1/2: samples of L1–L2 stage larvae; Larvae L3/4: samples of L3–L4 stage larvae; pupae: samples of pupae; Male_aeg, males of *Ae. aegypti*; Female_aeg, females of *Ae. aegypti*; and Female_alb, females of *Ae. albopictus*.

Bacterial community structure was observed through beta diversity analysis and looking at *Ae. aegypti* and those present in immature stages, as well as the water from artificial breeding sites (PERMANOVA F-value = 2.60; *p*-value = 0.001; R^2^ = 0.43). This differentiation was further confirmed by NMDS analysis (Figure 9B) which revealed two distinct groups: one group consisting of adult *Ae. aegypti* and females of *Ae. albopictus*, and a second group associated with the immature stages and water samples. Both analyses demonstrated significant separation between the two groups (PERMANOVA F-value = 2.60; *p*-value = 0.001; R^2^ = 0.43 and a stress level of 0.19).

Overall, we observed two distinct groups of bacterial communities, with one group comprising the ASVs of adults of *Ae. aegypti* and *Ae. albopictus*, separated from the sequences corresponding to water samples, larvae, and pupae. This significant difference (*p*-value = 0.001) confirms that the bacterial communities in adults were different from those found in immature stages, although the latter were similar to those in the bacterial communities of water from *Ae. aegypti*’s breeding sites.

In agreement with the previously observed results, the heatmap highlighted significant differences in the abundance and bacterial community structure of artificial breeding sites, water, immature *Aedes*, and adults of *Ae. aegypti*. The adults were strongly correlated with the genera *Asaia*, *Cedecea*, and *Elizabethkingia*, which were specific to the adults and did not have a high abundance in the other developmental stages or the bacterial community of the breeding site water (Figure S4). In contrast, water samples from different artificial breeding sites, as well as Larvae L1–L2, L3–L4, and pupae, exhibited high variation in their bacterial community structure. Notably, some genera, such as *Acinetobacter*, *Dechloromonas*, *Enterococcus Flectobacillus*, *Leifsonia*, *Stenotrophomonas*, and *Microbacterium*, were present in two or more developmental stages, evidencing the relationship between their bacterial communities (Figure S4).

**Figure 10:**
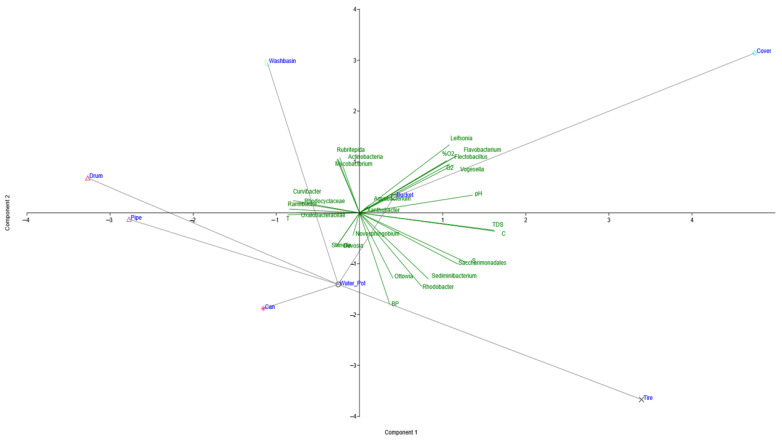
Multivariate analysis (PCA) of the influence of the bacterial community and physicochemical variables on the different types of artificial breeding sites. BP: barometric pressure, TDSs: total dissolved solids, O_2_: dissolved oxygen concentration, %O_2_: percentage of dissolved oxygen, S: salinity, C: conductivity, T: temperature. Types of breeding sites (blue) and physicochemical and biological variables (green).

**Figure 11:**
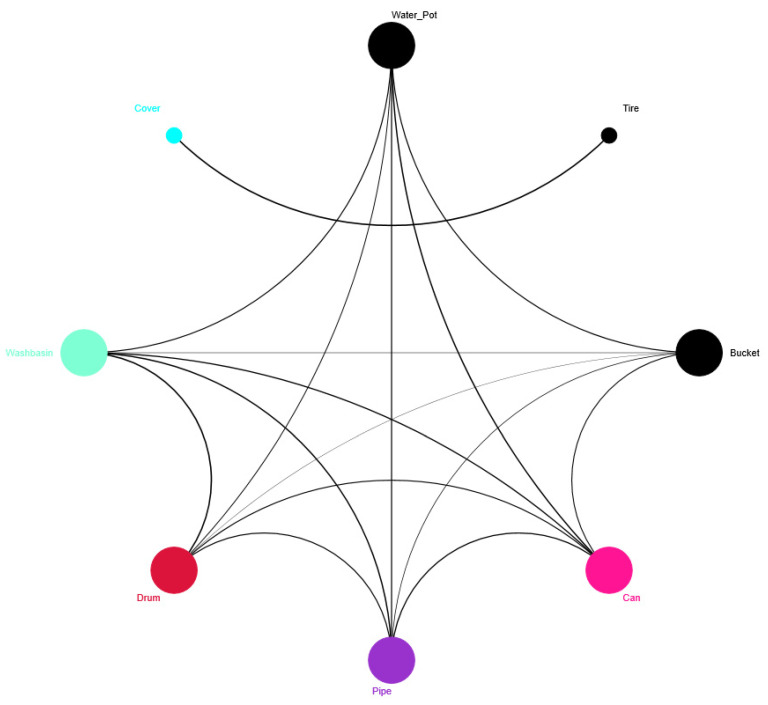
Correlation of the 20 most abundant bacterial genera and the influence of the physicochemical parameters associated with each type of breeding site based on the Bray–Curtis index.

Finally, multivariate analysis assessing the interaction between different artificial breeding sites for *Ae. aegypti*, physicochemical variables, and the most abundant bacterial genera in the water bodies showed an influence of bacterial communities on the different breeding site types. This analysis generated three distinct groups: The first group comprised washbasins, drums, and pipes (Figure 10 and Figure 11), which were associated with high temperature and low salinity and showed a high abundance of *Rhodocyclaceae*, *Ramilibacter*, *Actinobacteria*, and *Mycobacterium* (Figure 10). The second group included buckets, plant pots, and cans (Figure 10) and was related to genera such as *Xanthobacter*, *Aquabacterium*, *Novosphingobium*, and *Ottowia* (Figure 10). The third group was associated with the tire and drum cover breeding sites (Figure 10 and Figure 11), which exhibited high levels of total dissolved solids, conductivity, and salinity, and included genera such as *Flectobacillus*, *Leifsonia*, *Novosphingobium*, *Ottowia*, and *Rhodobacter* (Figure 10). All of these findings are summarized in the interaction network of *Ae. aegypti* artificial breeding sites (see Figure 11). This network illustrates that the tire and drum cover breeding sites are directly related and distinct from other interactions, while the other container types show multiple correlations among themselves. Notably, the plant pot breeding site demonstrates strong correlations (indicated in black) with all other types of breeding sites.

## 4. Discussion

The present study provided new information on the bacterial community associated with water in artificial breeding sites of *Ae. aegypti* in Leticia, Amazonas. It explored the correlation between this bacterial community and selected physicochemical variables that may serve as bioindicators for characterizing these breeding sites. Through the methodology proposed in this research, a significant interaction was found between temperature, salinity, and the high abundance of bacteria, including *Acinetobacter*, *Novosphingobium*, and *Ottowia* in various breeding sites such as washbasin, drum, pipe, tire, and drum cover.

Additionally, the bacterial communities present through the stages of *Ae. aegypti* from the Amazon were described. It was found that several ASVs, such as *Elizabethkingia*, *Acinetobacter*, *Chryseobacterium*, and *Aquabacterium*, might pass through all stages of mosquito development. Interestingly, a more specialized bacterial community was observed in adults, primarily consisting of *Elizabethkingia*, *Cedecea*, and *Asaia*, which differed from the communities found in waters and immature stages.

The most abundant identified positive breeding sites in the study area were buckets, tires, and plant pots. These sites have been widely reported as prevalent breeding locations in other research as well [28,64,65,66,67]. They have been found in both urban and rural areas, specifically in intra- and peridomicile houses. Independently of the climatic variability and geography of the study area, *Ae. aegypti* consistently shows a preference for these types of breeding sites.

Another study conducted in urban areas of India found that preferred breeding sites for mosquitoes were mainly clay. However, reports were found in other plastic containers, since they were the most accessible for the vectors in the area. These findings are consistent with other reports and highlight the adaptability of *Ae. aegypti*, as it can thrive in diverse breeding sites. This demonstrates its rapid ability to adjust to available water bodies and the physicochemical conditions present in those environments [68,69,70].

At this point, it is important to mention that the water stress in Leticia, Amazonas, characterized by high temperatures and low rainfall during the collection period [53,54], could have influenced the selection of some types of breeding sites by mosquitoes. Specifically, breeding sites such as pipes and cans, which held rainwater, were exposed to environmental oxidation, direct sunlight, high levels of suspended residues, and high temperatures. Temperature is one of the most critical physicochemical variables that impact the life cycle development of *Ae. aegypti*, as has been well documented [71,72,73,74]. This is particularly important because higher temperatures significantly reduce the time it takes for larvae development. Artificial breeding sites for various mosquito species, including culicids and anophelines, have reported a wide range of water temperatures, from 9.8 °C to 31.5 °C [29,75,76]. However, the increase in the average global temperatures of the planet has caused the water bodies of artificial breeding sites to have a temperature greater than 27 °C, reaching temperatures of up to 38 °C, where larval stages have been found [24,30]. This same behavior was found in our study, where the average temperature of the different types of breeding sites was 29 °C and the maximum temperature was 33 °C, associated with pipes.

Additionally, other research has found that the increase in temperature and the high availability of food favors the female/male ratio for *Ae. aegypti* and other species of the same genus [77,78,79]. In this sense, the increase in temperature could raise the risk of contracting dengue and other arboviral diseases in endemic areas due to the hematophagous activity of females and should be considered in the aedic indices, niche models, or potential distribution models to generate comprehensive vector control strategies [25,77].

Likewise, the high-salinity values found mainly in tires and the drum cover can be explained by considering water evaporation, the material of the container, and the presence of possible leachates that can alter the salt concentration in the breeding sites [24]. Furthermore, another study conducted by Medeiros-Sousa [75], where a prediction model was proposed for the presence and abundance of immature *Ae. aegypti* and *Ae. albopictus*, implementing physicochemical variables such as dissolved oxygen, pH, type of container, and salt concentration in the breeding water, also found that both species tolerate wide ranges of salinity. It should be underscored that in our study, the other physicochemical characteristics were constant in the different types of breeding sites and could be considered ecological markers (bioindicators). However, more in-depth studies ought to be conducted, considering systematic monitoring to confirm this, because the comparative analysis with the negative water body showed no difference in the physicochemical characteristics between positive and negative breeding sites (Figure S1).

Regarding the biological variables and in agreement with other studies, the most abundant phyla of the bacterial communities of both the water and different developmental stages of *Ae. aegypti* were Proteobacteria, Bacteroidota, Actinobacteria, and Firmicutes, as has been reported for *Ae. aegypti* previously [38,80,81,82]. It is noteworthy that some phyla were more specific for the water breeding samples, such as Verrucomicrobia and Cyanobacteria, which have also been reported in water bodies from *Ae. aegypti* and *Ae. albopictus* breeding sites [27,42,80]. Likewise, larvae present a greater richness of phyla, given that, at this stage of development, the greatest amount of food must be used, for which they must have the capacity to metabolize different food sources and the bacteria in the midgut could improve these processes.

In contrast, *Ae. aegypti* adults obtained from immature series showed a higher abundance of Bacteroidota followed by Proteobacteria, compared to previous reports that recorded a higher predominance of Firmicutes and Actinobacteria [35,82]. These changes in higher taxonomic levels suggest that adults could present specific bacterial communities that allow them to process different types of diet, which can change the conditions of the digestive tract such as pH, reactive oxygen species (ROS), and interaction with the insect immune system [38,83].

A similar pattern was observed at the genus level; the water bodies showed high diversity and dominance by *Xhantobacter*, *Rhodocyclaceae (C39)*, and *Novosphingobium*, which have been previously reported in water samples from artificial breeding sites of *Ae*. *aegypti* and *Ae*. *albopictus* in dengue-endemic areas of Thailand, French Guiana, and Guadeloupe [24,80]. In addition, a significant presence of *Ottowia* and *Leifsonia* was found, which had not been reported in *Ae. aegypti* breeding site waters, but in wastewater bodies, breeding sites of *Anopheles darlingi*, larvae, and adults of *Aedes* spp. [27,84,85,86].

These bacterial genera have been reported for their capacity to biodegrade aromatic organic compounds favoring nitrogen and carbon fixation in water. In our study, these genera were also found in immature states with a low relative abundance. Additionally, genera such as *Aquabacterium*, *Acinetobacter*, *Chryseobacterium*, *Dechloromonas*, and *Flectobacillus* were found, which were the most abundant at this developmental stage. Nevertheless, despite their reports on larvae and adults of *Ae. aegypti* and *Ae*. *albopictus*, information on the role they could play in larval development and the water bacterial community from mosquito breeding sites is limited; even so, it is believed that these bacterial genera were acquired through feeding habits [24,27,80].

Within this group of bacteria, *Chryseobacterium* and *Acinetobacter* stand out for the roles that they play in the insect, mainly due to the production of hydrolytic enzymes (proteases, lipases, and amylases) that allow the degradation of organic compounds and promote the absorption of nutrients in larval and adult stages, as has been reported for other insects [87,88]. In addition, these genera have been reported as producers of antimicrobial compounds, being determinants in the modulation of bacterial communities [84,86,89].

On the contrary, the genera *Aquabacterium*, *Dechloromonas*, and *Flectobacillus* are more related to bioremediation and water decontamination biotechnology application, processes that can be decisive for the development of immature organisms in conditions without high chlorine content or toxic compounds that can influence the development of eggs, larvae, and pupae [90,91,92,93]. The bacterial communities of artificial breeding site water demonstrated a close relationship with the physicochemical characteristics of the water.

*Flavobacterium* and *Flectobacillus* genera exhibited a correlation with the bucket and drum cover breeding site, which presented high levels of dissolved O_2_ and pH above 7.5, optimal conditions for their growth in freshwater [90,94]. Meanwhile, *Ottowia* and *Rhodobacter* have been previously isolated in wastewater with high levels of total dissolved solids [95,96,97]. These genera present aerobic and anaerobic metabolism for nitrifying and denitrifying capacity to reduce the total dissolved solids of wastewater. It is important to highlight that these two bacterial genera were found in water from artificial breeding sites associated with tires which have high levels of TDS, salinity, and conductivity. In contrast, *Novosphingobium* presented a high prevalence in the analyzed water bodies, mainly due to its ubiquity since it has been found in a wide variety of habitats such as soil, coastal sediments, freshwater sediments, lakes, and wastewater, as well as in association with plants and *Aedes* breeding sites [95,98].

Likewise, a group of bacterial genera consisting of *Rhodocyclaceae (C39)*, *Curvibacter*, and *Ramilibacter* were found in the washbasin, drum, and pipe breeding sites, but they were not related to the physicochemical profile of the artificial breeding sites. However, they were associated with bioremediation and decontamination processes of toxic compounds, being considered as bacteria with a high biotechnological potential [98,99,100].

Another finding was the presence of *Mycobacterium* in the bacterial community of water bodies associated with the washbasin. This bacterial genus is considered important in public health since reports have been found of a group of non-tuberculous mycobacteria (NTM), which are transmitted by contaminated water and might generate acute respiratory conditions in humans [101,102]. Similarly, in water and larvae samples, there were low abundances of bacterial genera associated with coliforms and other intestinal diseases such as *Escherichia/Shigella*, *Salmonella*, *Enterobacter*, *Clostridium* spp., and *Citrobacter* (Table S7) [103,104]. These findings are of significant relevance, particularly considering that the water where genera were found is used by the community in the absence of drinking water in the region, which represents a high risk to human health.

The composition profile of the adults’ bacterial communities differed from that found in water and immature stages, possibly associated with the final metamorphosis process and the loss of many ASVs during the passage from pupa to the adult stage. Additionally, it should be taken into consideration that the diets of immature and adults differ, which may have affected the composition of the bacterial communities of the different stages, particularly because microorganisms in the midgut specialize in obtaining nutrients from different carbon sources.

Also, these changes in microbiome composition are presented in laboratory and field-collected mosquitoes, due to the feeding habits that enhance chemical and biological changes in the lumen midgut, favoring specific and conserved bacterial communities [105,106].

In the present study, the bacterial community of the adults obtained from the immature series was mainly composed of the genera *Elizabethkingia* and *Asaia*, previously reported in the midgut and sexual organs of *Ae. aegypti* and *Ae. albopictus* laboratory populations [36,38,39]. The genera *Elizabethkingia* and *Asaia* play a crucial role in the life cycle of the vector since they have been reported to accelerate the life cycle of *Ae. aegypti* [107,108]. In addition, genes associated with antibiotic resistance and blood degradation have been found in *Elizabethkingia*, which would allow it to modulate the bacterial communities of the breeding site and the midgut of the insect [107,109,110].

Furthermore, studies in *Anopheles*, *Aedes*, and other culicids have demonstrated a competitive relationship between *Asaia* spp. and *Wolbachia* spp. Both of these bacteria have been present in these insects, but one often dominates over the other [111,112]. These complex relationships remain the focus of new studies, which allow us to understand the transmission dynamics of arboviral diseases and enhance control methods.

Another bacteria genus identified in high abundance in adults was *Cedecea*. This genus, although considered rare, has been found in insecticide-resistant *Aedes* spp. and *Anopheles*, as well as *Acinetobacter*, *Aquabacterium*, and *Chryseobacterium*, indicating that these genera could play an important role in insecticide resistance and degradation [34,113,114,115]. In addition, other studies have shown that the presence of both *Cedecea* and *Serratia* in the microbiota of *Aedes* spp. and *Culex* can interfere with establishing *Asaia* and *Pseudomonas* [116].

In addition, a group of bacteria that deserves special attention comprises *Acinetobacter*, *Aquabacterium*, *Chryseobacterium*, and *Elizabethkingia*, all of which were found at various developmental stages and in breeding water, suggesting the possibility of trans-stadial transmission. Studies on populations of *Ae. aegypti* have shown that genera such as *Acinetobacter*, *Chryseobacterium*, and *Elizabethkingia* are abundant in adults and can be transmitted from females to the breeding site water on the surface of eggs [43,107,117]. This ensures that the aquatic habitat maintains an adequate bacterial structure for the development of larvae.

Finally, there was incidental detection of ASVs (Amplicon Sequence Variants) linked with archaea in water samples from breeding sites (Table S8), as well as in larvae and pupae. Taxonomic groups associated with methane production, such as *Methanogenium*, *Methanosarcina*, *Methanobacterium*, and *Methanobrevibacter*, along with extremophiles associated with ammonia oxidation (Candidatus *Nitrosotalea* and Candidatus *Nitrososphaera*) and organisms tolerant to high salinity (*Halococcus* and *Halorubrum*) and high temperatures (*Thermococcus aggregans*), were observed. These findings are of considerable importance to the ecology of water and vector populations, as the microorganisms identified may alter the physicochemical conditions of the water, oxidizing toxic compounds like ammonia and contributing to the adaptation of bacterial communities in saline environments. Furthermore, the presence of these microorganisms in the immature stages of *Ae. aegypti* could facilitate adaptation to new environmental conditions for breeding insect vectors. This adaptability allows for an expansion of their habitat, increases their resilience to climate change, and raises the risk of arboviral disease transmission. However, further research is needed to clarify the roles of these microbial groups.

## 5. Conclusions

The analysis of the diversity of the different breeding sites and the physicochemical profile demonstrated the environmental plasticity of *Ae. aegypti*, mainly influenced by the temperature and salinity of the water bodies. Additionally, the presence of *Aquabacterium*, *Dechloromonas*, *Flectobacillus*, *Leifsonia*, and *Ottowia* within the water bodies of the breeding sites is related to bioremediation processes, which may be playing a role in the suitability of the water bodies for the breeding of immature stages.

In this context, some bacterial genera such as *Acinetobacter*, *Aquabacterium*, *Chryseobacterium*, and *Elizabethkingia* can be transmitted from the water of breeding sites to the adult stage, suggesting a trans-stadial passage during the life cycle.

Adding to this information, the structure of the bacterial community of *Ae. aegypti* adults, which was dominated by *Elizabethkingia*, *Cedecea*, and *Asaia*, significantly differs from that found in the immature *Aedes* and water bodies associated with artificial breeding sites in the Amazon of Colombia.

Likewise, in the water bodies, incidental detection of bacterial genera associated with bacteria of importance in public health such as *Escherichia*, *Salmonella*, *Enterobacter*, *Mycobacterium*, *Clostridium*, and *Citrobacter* was made, which can represent a public health problem for the inhabitants of Leticia, Amazonas.

## Data Availability

Sequence data associated with the 16S rRNA gene amplicon reads are available in the SRA database under the Bioproject code PRJNA1185382.

## Supplementary Materials

The following supporting information can be downloaded at https://www.mdpi.com/article/10.3390/insects16020195/s1: Figure S1: Graphical representation of the average physicochemical characteristics of positive and negative artificial breeding sites grouped by Tukey’s statistics; Figure S2: Rarefaction curve of the bacterial communities of artificial breeding site water, immature *Aedes*, and adults; Figure S3: Pattern search using Pearson correlation coefficient (r) of the most important bacterial genera with the highest correlations; Figure S4: Heatmap of bacterial communities in water from artificial breeding site water, *Ae. aegypti* larvae, pupae, and adults, and *Ae. albopictus* females; Table S1. Inventory of positive and negative artificial breeding sites for *Ae. aegypti* explored in three neighborhoods of Leticia, Amazonas, collected in November 2023; Table S2. Groups of larvae, pupae, and adults of *Ae. aegypti* obtained from artificial breeding sites in Leticia, Amazonas; Table S3. Physicochemical profile of Ae. aegypti artificial breeding site from Leticia, Amazonas; Table S4. Water samples from artificial breeding sites, *Ae. aegypti* larvae, pupae, and adults, and *Ae. albopictus* females were selected for total DNA extraction, 16S rRNA gene amplification, and 16S rDNA V3-V4 region sequencing by Illumina Miseq; Table S5. Relative abundance of bacterial genera found in water from artificial breeding sites and all developmental stages of *Ae. aegypti*; Table S6. Bacterial genera that correlate most strongly with the genus *Asaia* in water samples from artificial breeding sites and sites of immature and adult *Ae. aegypti*, based on Pearson correlation (r); Table S7. Relative abundance of coliform-associated sequences and public health interest bacteria related to intestinal diseases in humans found in samples from artificial breeding facilities, larvae, and adults of *Ae. aegypti*; Table S8. Count of reads associated with archaea sequences in samples from artificial breeding sites and larvae of *Ae. aegypti.*
