# Supplementary material for: Study of Bacterial Communities in Water and Different Developmental Stages of Aedes aegypti from Aquatic Breeding Sites in Leticia City, Colombian Amazon Biome
Source: Insects. 2025 Feb 11;16(2):195. doi: 10.3390/insects16020195 (PMC11856942; doi:10.3390/insects16020195)
Supplement: Supplementary file 1 [file insects-16-00195-s001.zip › Figures S1-S4.pdf]

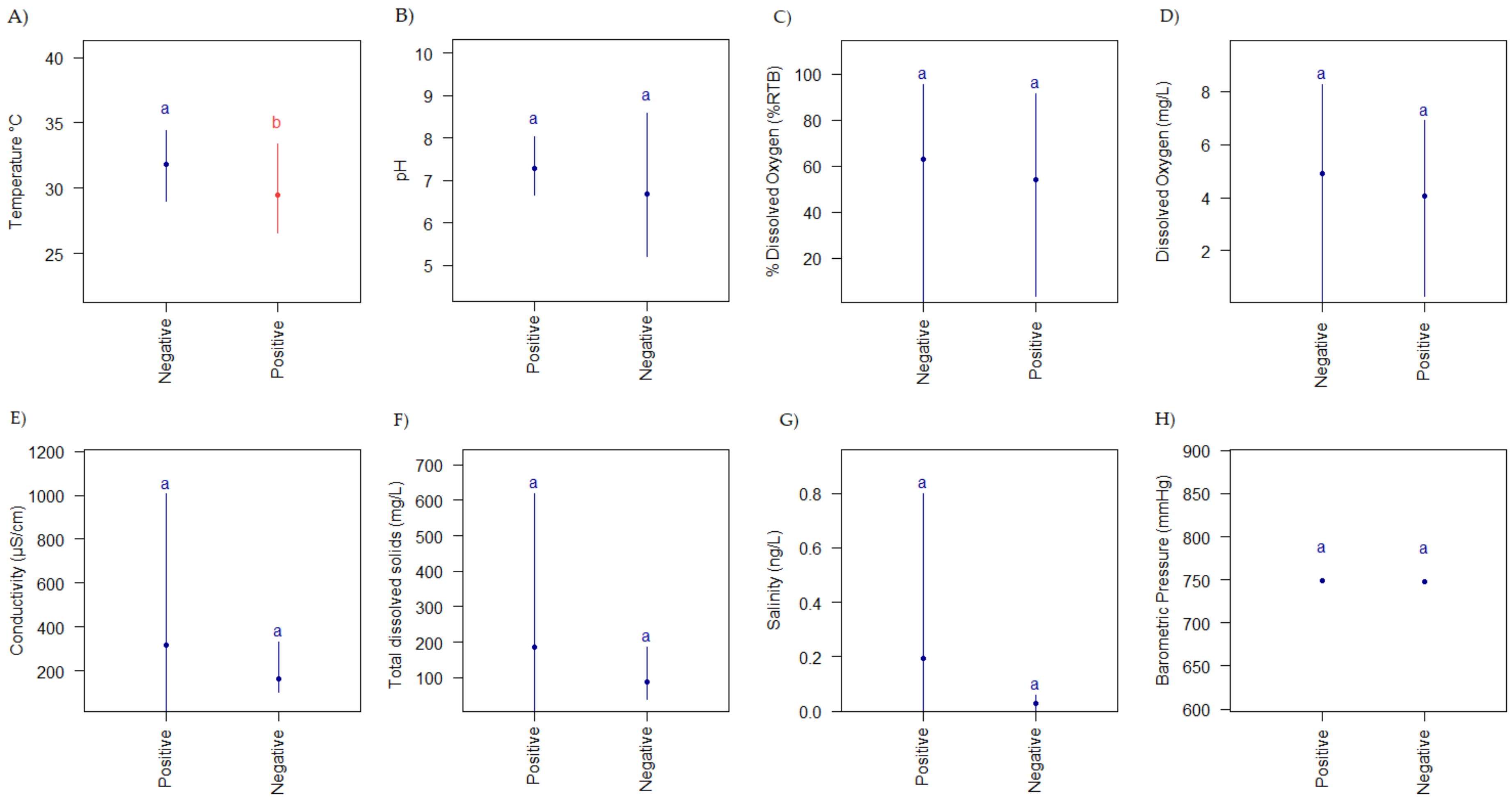


**Figure S1.** Graphical representation of the average physicochemical characteristics of positive and negative artificial breeding sites grouped by Tukey's statistic **A)** Temperature (P-value = 0.019), two groups (a and b) are shown that are significantly different, **B)** pH (P-value = 0.17), **C)** Percentage of dissolved oxygen (P-value = 0.504), **D)** Concentration of dissolved oxygen (P-value = 0.435), **E)** Electrical conductivity (P-value = 0.242), **F)** Total dissolved solids (P-value = 0.23), **G)** Salinity (P-value = 0.111, **H)** Barometric pressure (P-value = 0.449).

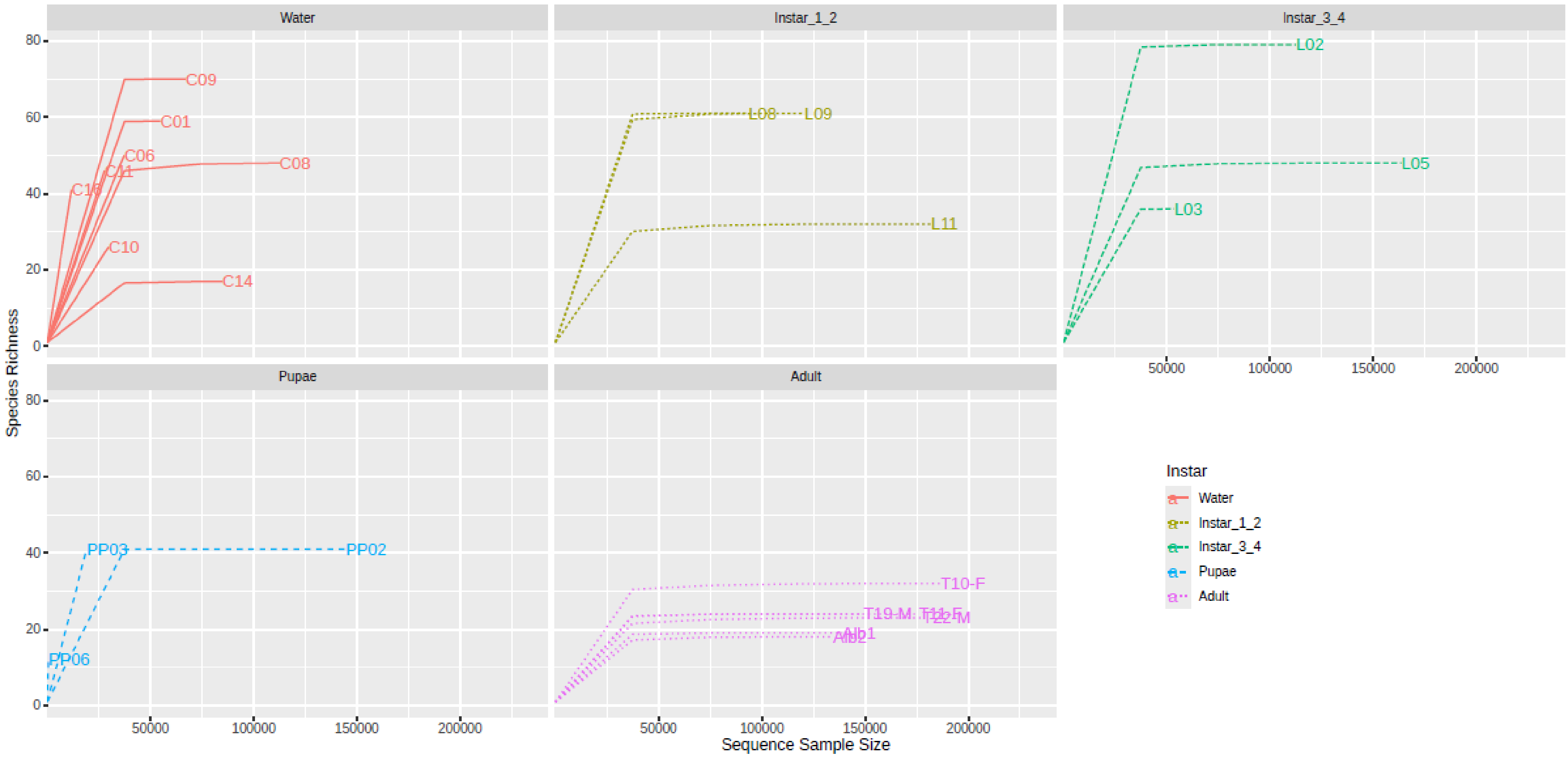


**Figure S2.** Rarefaction curve of the bacterial communities of artificial breeding site water, immature *Aedes*, and adults. Artificial breeding site water (C01-C16), L1/L2 larvae (L08—L11), L3/L4 larvae (L02-L05), pupae (PP02-PP06), *Ae. aegypti* males (T19_M and T22_M), *Ae. aegypti* females (T10_F and T11_F), and *Ae albopictus* females (Alb1 and Alb2)

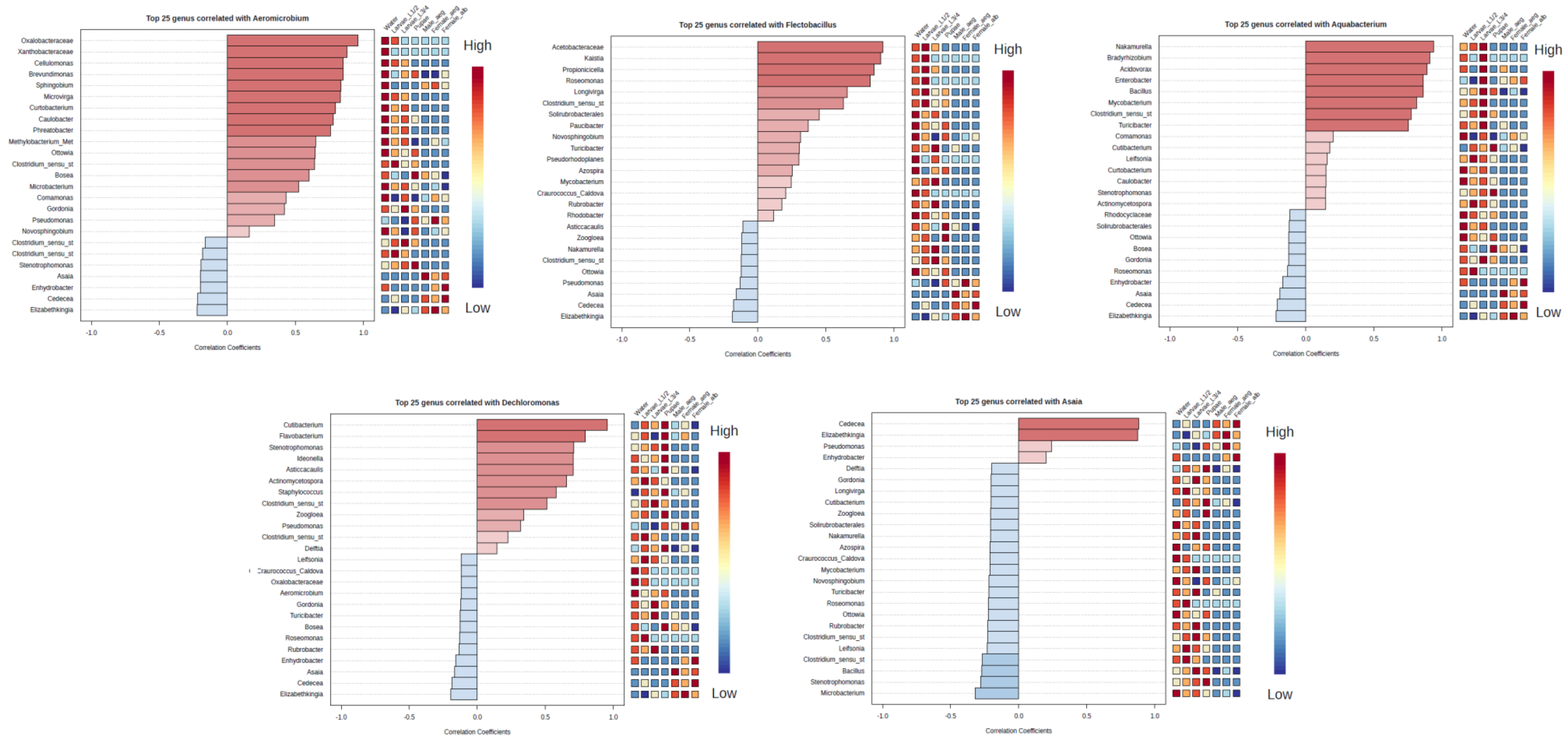


**Figure S3.** Pattern search using Pearson correlation coefficient (r) of the most important bacterial genera with the highest correlations. Positive and negative corrections are represented in red and blue, respectively. The color scale from deep red to dark blue indicates the intensity of the correlation associated with the different development stages or water samples.

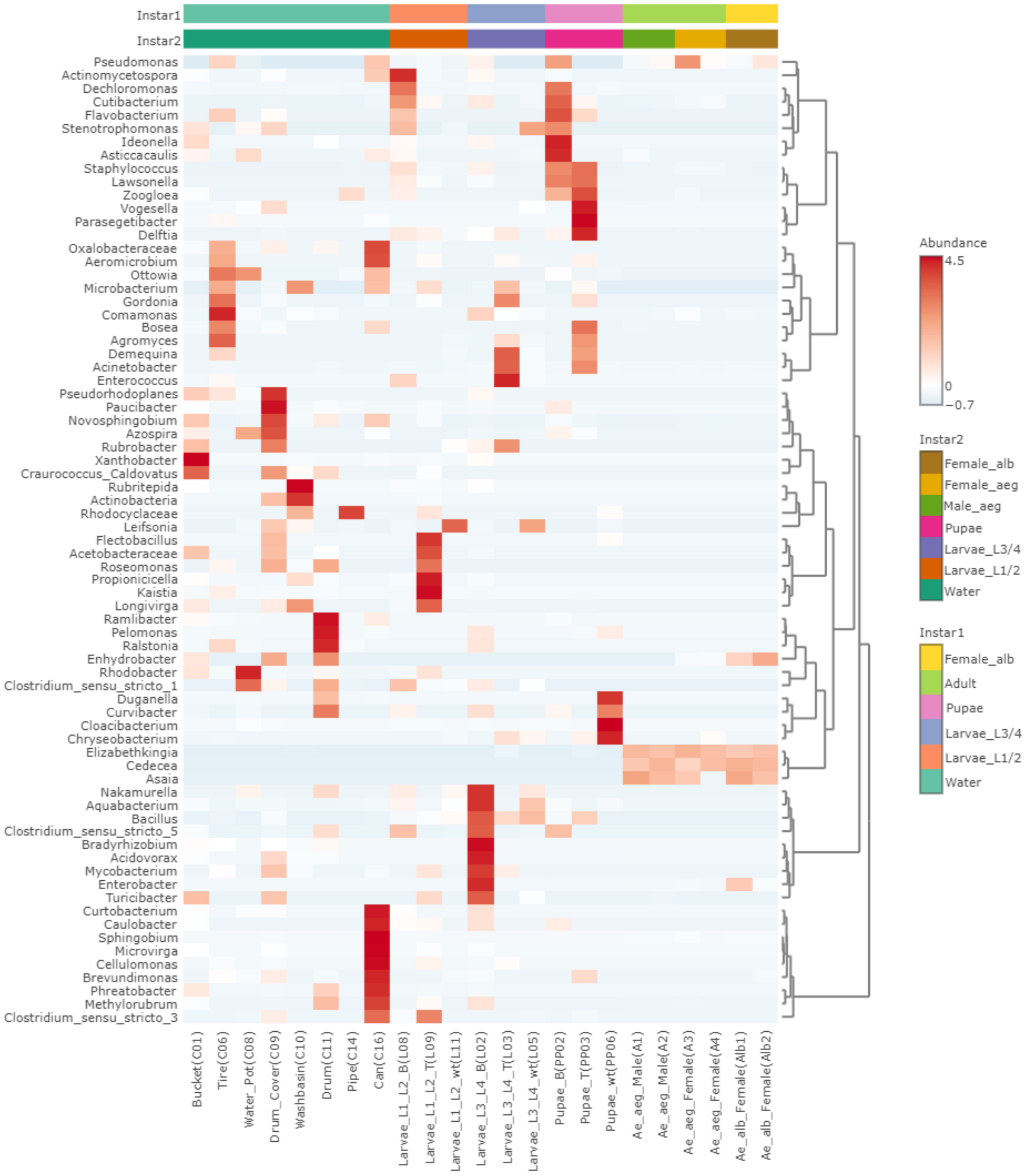


**Figure S4.** Heatmap of bacterial communities in water from artificial breeding sites water, *Ae. aegypti* larvae, pupae and adults, and *Ae. albopictus* females.
