# Supplementary material for: Study of Bacterial Communities in Water and Different Developmental Stages of Aedes aegypti from Aquatic Breeding Sites in Leticia City, Colombian Amazon Biome
Source: Insects. 2025 Feb 11;16(2):195. doi: 10.3390/insects16020195 (PMC11856942; doi:10.3390/insects16020195)
Supplement: Supplementary file 1 [file insects-16-00195-s001.zip › Tables S1-S8.pdf]

Table S1. Inventory of positive and negative artificial breeding sites for *Ae. aegypti* explored in three neighborhoods of Leticia, Amazonas collected in November 2023.

Type of Breeding Site	Code	DNA Concentration (ng/μL)	Neighborhood	Inmanture detection
Washbasin	C10	9.03	Porvenir	Positive
Bucket	C01	21.85	Once de Noviembre	Positive
Bucket	C07	6.23	Once de Noviembre	Positive
Bucket	C13	11.55	Once de Noviembre	Positive
Drum	C11	10.28	Porvenir	Positive
Can	C16	8.33	Colombia	Positive
Tire	C06	14.33	Once de Noviembre	Positive
Tire	C04	15.65	Once de Noviembre	Positive
Plant Pot	C15	26.80	Once de Noviembre	Positive
Plant Pot	C08	15.63	Once de Noviembre	Positive
Drum Cover	C09	11.95	Porvenir	Positive
Pipe	C14	13.95	Porvenir	Positive
Bucket	C17	-	Colombia	Negative
Pipe	C18	-	Colombia	Negative
Drum	C19	-	Once de Noviembre	Negative
Drum	C20	-	Once de Noviembre	Negative
Drum	C21	-	Once de Noviembre	Negative
Street hole	C22	-	Porvenir	Negative

Table S2. Groups of Larvae, pupae, and adults of *Ae. aegypti* obtained from artificial breeding sites in Leticia, Amazonas

***Ae. aegypti* developmental stage**	**Code**	**Number of individuals per sample**	**DNA concentration (ng/µl)**	**Source**
Larvae_L1-L2_B	L7	2	6,60	Bucket
Larvae_L1-L2_B	L8	5	7,40	Bucket
Larvae_L1-L2_T	L9	3	26,95	Tire
Larvae_L1-L2_T	L10	5	16,65	Tire
Larvae_L1-L2_P	L11	4	7,55	Plant Pot
Larvae_L3-L4_B	L1	5	10,90	Bucket
Larvae_L3-L4_B	L2	5	3,85	Bucket
Larvae_L3-L4_T	L3	5	6,20	Tire
Larvae_L3-L4_T	L4	4	4,67	Tire
Larvae_L3-L4_P	L5	1	9,65	Plant Pot
Larvae_L3-L4_P	L6	6	7,25	Plant Pot
Pupae_B	PP1	1	17,38	Bucket
Pupae_B	PP2	5	17,60	Bucket
Pupae_T	PP3	5	26,13	Tire
Pupae_T	PP4	1	9,53	Tire
Pupae_P	PP5	2	21,08	Plant Pot
Pupae_P	PP6	1	16,18	Plant Pot
Male_1	A1	6	649,80	-
Male_2	A2	3	337,60	-
Female_1	A3	6	139,98	-
Female_2	A4	5	82,33	-

Table S3. Physicochemical profile of Ae. aegypti artificial breeding site from Leticia, Amazonas.

Breeding site	Code	Temperature	pH	Barometric pressure	O2 Percentage	O2 Concentration	Conductivity	Total dissolved solids	Salinity	Neighborhood	Immature detection
Washbasin	C10	28.9	7.5	747.3	59	4.44	122.1	73	0.05	Porvenir	Positive
Bucket	C01	26.6	6.9	750.3	61.3	4.7	102.1	63	0.04	Once de Noviembre	Positive
Bucket	C07	29.1	7.3	750	54.4	4.13	249.2	150	0.11	Once de Noviembre	Positive
Bucket	C13	29.1	7.5	747.2	56.8	4.28	727	438	0.32	Porvenir	Positive
Drum	C11	29.6	6.9	747	43.2	3.22	19.2	11	0.01	Porvenir	Positive
Can	C16	29.7	7.3	749.7	39.7	2.96	192.4	115	0.08	Colombia	Positive
Tire	C05	28.1	8	750.3	61.4	4.72	1,006	618	0.46	Once de Noviembre	Positive
Tire	C04	30.3	6.9	750.6	50.2	3.72	195.9	116	0.8	Once de Noviembre	Positive
Plant pot	C15	29.4	6.7	750.3	91.5	6.89	115	7	0	Once de Noviembre	Positive
Plant pot	C08	29.4	7.1	749.8	3.6	0.27	346.6	208	0.15	Once de Noviembre	Positive
Drum cover	C09	29.6	7.9	747.33	77	5.68	579	345	0.25	Porvenir	Positive
Pipe	C14	33.4	7.4	747	53.8	3.77	150.4	84	0.06	Porvenir	Positive
Bucket	C17	29	7.6	749.7	95.4	7.22	144.2	87	0.06	Porvenir	Negative
Pipe	C18	34.4	6.3	748.2	1.3	0.09	166.1	92	0.04	Colombia	Negative
Drum	C19	29.6	5.3	748.2	55.1	4.16	103.4	62	0	Colombia	Negative
Drum	C20	30.9	5.2	748.1	63.2	4.32	112.3	53	0.01	Once de Noviembre	Negative
Drum	C21	33.3	7.1	748.1	76.2	5.34	123.2	39	0	Once de Noviembre	Negative
Street hole	C22	33.6	8.6	748	87.7	8.25	330.2	187	0.06	Once de Noviembre	Negative
Average		30.2	7.1	748.7	57.3	4.3	265.8	152.7	0.1	-	-
F-value		4.0	1.9	3.1	0.6	1.0	0.9	1.0	5.8	-	-
P-value		0.0279	0.171	0.0559	0.7290	0.4860	0.5300	0.5140	0.0084	-	-

Table S4. Water samples from artificial breeding sites, *Ae. aegypti* larvae, Pupae and adults, and *Ae. albopictus* females were selected for total DNA extraction, 16S rRNA gene amplification, and 16S rDNA V3-V4 region sequencing by Illumina Miseq.

Name	Code	Type of sample	No. of individual	DNA concentration (ng/µl)	No. of reads (filtrated)	Source	Species
Washbasin	C10	Water	-	9.03	118,358	Porvenir	*Ae. aegypti*
Bucket	C01	Water	-	21.85	103,308	Once de noviembre	*Ae. aegypti*
Drum	C11	Water	-	10.28	56,678	Porvenir	*Ae. aegypti*
Can	C16	Water	-	8.33	105,933	Colombia	*Ae. aegypti*
Tire	C06	Water	-	14.33	104,900	Once de noviembre	*Ae. aegypti*
Plant pot	C08	Water	-	15.63	147,231	Once de noviembre	*Ae. aegypti*
Drum cover	C09	Water	-	11.95	104,859	Porvenir	*Ae. aegypti*
Pipe	C14	Water	-	13.95	120,367	Porvenir	*Ae. aegypti*
Larvae_L1/L2_B	L8	*Larvae_L1/L2*	5	7.40	160,433	Bucket	*Ae. aegypti*
Larvae_L1/L2_T	L9	*Larvae_L1/L2*	5	26.95	96,848	Tire	*Ae. aegypti*
Larvae_L1/L2_P	L11	*Larvae_L1/L2*	5	7.55	184,616	Plant pot	*Ae. aegypti*
Larvae_L3/L4_B	L2	*Larvae_L3/L4*	5	3.85	135,961	Bucket	*Ae. aegypti*
Larvae_L3/L4_T	L3	*Larvae_L3/L4*	5	6.20	147,478	Tire	*Ae. aegypti*
Larvae_L3/L4_P	L5	*Larvae_L3/L4*	1	9.65	169,505	Plant pot	*Ae. aegypti*
Pupae_B	PP2	*Pupae*	5	17.60	179,531	Bucket	*Ae. aegypti*
Pupae_T	PP3	*Pupae*	5	26.13	174,716	Tire	*Ae. aegypti*
Pupae_P	PP6	*Pupae*	1	16.18	30,723	Plant pot	*Ae. aegypti*
Male_1	A1	Male	6	649.80	175,485	-	*Ae. aegypti*
Male_2	A2	Male	3	337.60	178,954	-	*Ae. aegypti*
Female_1	A3	Female	6	139.98	177,546	-	*Ae. aegypti*
Female_2	A4	Female	5	82.33	188,684	-	*Ae. aegypti*
Female_3	Alb1	Female	5	37.20	179,033		*Ae. albopictus*
Female_4	Alb2	Female	5	36.33	182,592	-	*Ae. albopictus*
Core community	CC	Control	-	35.63	128,781	-	-

Table S5. Relative abundance of bacterial genera found in water from artificial breeding sites and all developmental stages of *Ae. aegypti*

Genus	Relative abundance (%)					
	Water	Larvae L1-L2	Larvae L3-L4	Pupae	Male	Female
Aquabacterium	0,023%	0,093%	13,761%	6,857%	0,151%	0,783%
Acinetobacter	0,992%	4,614%	33,118%	2,210%	0,077%	0,022%
Chryseobacterium	0,012%	0,366%	3,283%	0,648%	0,043%	1,500%
Elizabethkingia	0,123%	0,002%	0,867%	0,335%	62,792%	66,529%

Table S6. Bacterial genera that correlate most strongly with the genus *Asaia* in water samples from artificial breeding sites, and sites of immature and adult *Ae. aegypti*, based on Pearson correlation (r)

Genus	Correlation	T-Stat	P-Value	FDR
Cedecea	0.84701	7.3017	3.45E-07	1.80E-05
Elizabethkingia	0.84154	7.139	4.86E-07	1.80E-05
Asaia	0.74476	5.1142	4.58E-05	0.0011295
Microbacterium	-0.426	-2.1578	0.042677	0.52468
Ottowia	-0.40076	-2.0045	0.058076	0.52468
Clostridium_sensu_stricto_1	-0.39883	-1.993	0.059411	0.52468
Craurococcus_Caldovatus	-0.39819	-1.9893	0.059856	0.52468
Oxalobacteraceae	-0.38352	-1.903	0.070838	0.52468
Novosphingobium	-0.36661	-1.8057	0.085315	0.52468
Roseomonas	-0.35221	-1.7246	0.099298	0.52468
Rhodococcus	-0.35013	-1.7129	0.10145	0.52468
Azospira	-0.33956	-1.6543	0.11293	0.52468
Pseudorhodoplanes	-0.33414	-1.6246	0.11917	0.52468
Pseudomonas	0.33198	1.6128	0.12171	0.52468
Actinomycetospora	-0.3318	-1.6118	0.12194	0.52468
Methylobacterium_Methylorubrum	-0.32536	-1.5768	0.12979	0.52468
Phreatobacter	-0.32241	-1.5608	0.13352	0.52468
Rhodobacter	-0.31611	-1.5269	0.14171	0.52468
Rhodocyclaceae	-0.31313	-1.5109	0.14571	0.52468
Longivirga	-0.3126	-1.5081	0.14643	0.52468
Solirubrobacterales	-0.30862	-1.4868	0.15192	0.52468
Aeromicrobium	-0.30573	-1.4715	0.15599	0.52468
Clostridium_sensu_stricto_3	-0.28665	-1.3712	0.1848	0.58608
Ralstonia	-0.28339	-1.3542	0.19008	0.58608
Ramlibacter	-0.27247	-1.2977	0.20847	0.59746
Rubrobacter	-0.26638	-1.2665	0.21922	0.59746
Gemmobacter	-0.2601	-1.2344	0.23068	0.59746
Xanthobacteraceae	-0.25731	-1.2202	0.2359	0.59746
Curtobacterium	-0.25483	-1.2077	0.24061	0.59746
Brevundimonas	-0.25025	-1.1845	0.24945	0.59746
Arsenicibacter	-0.24983	-1.1823	0.25029	0.59746
Cellulomonas	-0.24064	-1.1362	0.26869	0.60306
Caulobacter	-0.23681	-1.117	0.27663	0.60306
Rubritepida	-0.23394	-1.1026	0.28266	0.60306
Xanthobacter	-0.22599	-1.0631	0.29981	0.60306
Paucibacter	-0.22291	-1.0479	0.3066	0.60306
Leifsonia	-0.22267	-1.0467	0.30714	0.60306
Comamonas	-0.21995	-1.0332	0.31325	0.60306
Turicibacter	-0.21793	-1.0233	0.31783	0.60306

Pelomonas	-0.21037	-0.98611	0.3353	0.60907
Sphingobium	-0.20946	-0.98163	0.33746	0.60907
Propionicicella	-0.19002	-0.88692	0.38517	0.668
Flectobacillus	-0.18884	-0.88125	0.38816	0.668
Gordonia	-0.17084	-0.79457	0.43575	0.73286
Stenotrophomonas	-0.15572	-0.72243	0.478	0.76467
Mycobacterium	-0.15202	-0.70483	0.48866	0.76467
Kaistia	-0.15173	-0.70344	0.48951	0.76467
Bosea	-0.14827	-0.68705	0.49957	0.76467
Enterobacter	0.14596	0.67613	0.50633	0.76467
Nakamurella	-0.13736	-0.63547	0.53198	0.78734
Chryseobacterium	0.129	0.59611	0.55747	0.80888
Staphylococcus	0.12146	0.56076	0.58089	0.81699
Agromyces	-0.12011	-0.55443	0.58514	0.81699
Lawsonella	0.11196	0.51632	0.61103	0.8249
Curvibacter	-0.11131	-0.5133	0.6131	0.8249
Flavobacterium	0.10232	0.47134	0.64226	0.84869
Cloacibacterium	0.089946	0.41386	0.68317	0.86789
Acidovorax	-0.088908	-0.40905	0.68665	0.86789
Enhydrobacter	0.087322	0.4017	0.69197	0.86789
Clostridium_sensu_stricto_5	-0.080042	-0.36798	0.71657	0.88377
Bradyrhizobium	-0.074289	-0.34138	0.73621	0.88434
Parasegetibacter	0.068572	0.31498	0.75589	0.88434
Enterococcus	-0.062953	-0.28906	0.77537	0.88434
Delftia	0.06295	0.28905	0.77538	0.88434
Acinetobacter	0.062546	0.28718	0.77679	0.88434
Aquabacterium	-0.05176	-0.23751	0.81456	0.9045
Zoogloea	0.050519	0.2318	0.81894	0.9045
Asticcacaulis	-0.034921	-0.16013	0.87431	0.94518
Cutibacterium	0.027763	0.12728	0.89993	0.94518
Bacillus	-0.026549	-0.12171	0.90429	0.94518
Vogesella	0.025833	0.11842	0.90686	0.94518
Dechloromonas	-0.018249	-0.083642	0.93413	0.96008
Ideonella	0.013157	0.0603	0.95249	0.96553
Duganella	-0.0050736	-0.023251	0.98167	0.98167

Table S7. Relative abundance of coliform-associated sequences and public health interest bacteria related to intestinal diseases in humans found in samples from artificial breeding facilities, larvae, and adults of *Ae. aegypti*

Genus	Relative abundance (%%)			
	Water	Larvae	Pupae	Adults
Citrobacter	0,002%	-	-	-
Clostridium	1,622%	0,855%	0,515%	-
Enterobacter	0,010%	2,512%	0,037%	0,012%
Escherichia/ Shigella	0,003%	0,001%	-	0,005%
Salmonella	0,004%	-	-	0,006%

Table S8. Count of reads associated with Archaea sequences in samples from artificial breeding sites and larvae of *Ae. aegypti*

Source	Archaea	Reads
Drum_Cover(C09)	*Halococcus*	10
	Woesearchaeales 1	5
Drum(C11)	*Thermococcus aggregans*	3
	Halorubrum laminariae	2
Pipe(C14)	*Methanogenium frigidum*	6
	Methanosarcina barkeri	9
	Methanobacterium congolense	5
	Woesearchaeales 2	3
	Woesearchaeales 3	2
Larvae_L1_L2_B(L08)	*Methanobrevibacter arboriphilus*	4
	Candidatus_Nitrososphaera	4
Larvae_L3_L4_B(L02)	*Methanobacteriaceae 1*	93
	Methanobrevibacter arboriphilus	37
	Methanogenium frigidum	25
	Methanobacteriaceae 2	23
Pupae_p(PP06)	*Candidatus_Nitrosotalea 1*	17
	Candidatus_Nitrosotalea 2	16
